# Label‐Free Proteomic Profiling of the *dvls2* (CL2006) *Caenorhabditis elegans* Alzheimer's Disease (AD) Model Reveals Conserved Molecular Signatures Shared With the Human AD Brain

**DOI:** 10.1111/jnc.70152

**Published:** 2025-07-13

**Authors:** Iverson Conrado Bezerra, Emily Raphaely Souza dos Santos, Katarine G. Aurista do Nascimento, Artur José da Silva, Josivan Barbosa de Farias, Maria Luiza de Lima Vitorino, Roberto Afonso da Silva, José Luiz de Lima Filho, Priscila Gubert

**Affiliations:** ^1^ Keizo Asami Institute, iLIKA Federal University of Pernambuco Recife Brazil; ^2^ Graduate Program in Biology Applied to Health, PPGBAS Federal University of Pernambuco Recife Brazil

**Keywords:** Alzheimer, *C. elegans*, Conserved targets, Proteomics

## Abstract

Alzheimer's disease (AD) is the most common form of dementia, posing significant challenges to cognitive, emotional, social, and financial well‐being. The biochemical and molecular pathways associated with AD are complex, making it difficult to study and simulate in patients or through in vitro research. Thus, animal models play a crucial role in investigating the development and progression of AD. One widely used model in neuroscience studies is the free‐living nematode *Caenorhabditis elegans* ( *C. elegans* ). The development of transgenic animals has allowed for the construction of the *dvls2* (CL2006) *C. elegans* strain, which constitutively expresses the amyloid beta (Aβ) peptide. This study conducted a proteomic analysis on the *dvls2* (CL2006) strain. Also, a cross‐species comparative analysis was performed using microarray data from AD patients to identify genes with ontology in the *dvls2* (CL2006). A total of 543 proteins were found to be differentially regulated in the *dvls2* (CL2006) strain. Furthermore, in the analysis of the human datasets, 397 upregulated and 767 downregulated genes were identified. The differentially expressed genes (DEGs) were analyzed in Ortholist to identify their orthologs in *C. elegans* . Then, the orthologous genes in the *dvls2* (CL2006) model were compared to the proteomic data, resulting in the identification of 29 upregulated and 24 downregulated proteins (DEPs). Functional enrichment analysis of DEPs revealed terms related to pyruvate, glucose, and glutamate metabolism, in addition to binding activities to unfolded proteins and ligases, highlighting the upregulation of chaperone and ubiquitination‐associated proteins. Protein–protein network (PPI) was performed for the human DEGs and DEPs of *dvls2* (CL2006). Topological analyses of the networks were performed, revealing the following *C. elegans hub* proteins: EEF‐2, ALH‐13, ENOL‐1, RPL‐2, TPI‐1, CTS‐1, RPL‐9, RPL‐23, CCT‐1, and RPS‐8. eEF‐2 was identified as a key regulator of the human AD PPI and *dvls2* (CL2006). Modules were analyzed in the networks, and the presence of key regulators was identified. This study provides the first proteomic characterization of the AD model *dvls2* (CL2006) and a cross‐species comparative analysis with data from AD individuals, supporting the use of *dvls2* (CL2006) in AD studies.

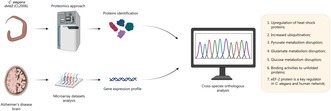

## Graphical abstract

Cross‐species analysis combining *C. elegans* proteomics and Alzheimer's disease brain transcriptomics reveals shared alterations in stress response, metabolism, and protein homeostasis, highlighting eEF‐2 as a conserved key regulator.

**Abbreviations**
AD: Alzheimer's disease
ALH: Aldehyde dehydrogenase
Aβ: β‐amyloid
BP: Biological process
BRCA: Breast cancer
*C*_*B*_
: Closeness betweenness
*C*_*C*_
: closeness centrality
CC: Cellular component
CCT‐1: Chaperonin‐containing TCP‐1
cDNA: Complementary DNA
Cys: Cysteine
DEGs: Differentially expressed genes
DEPs: Differentially expressed proteins
eEF‐2: Elongation factor 2
EEF‐2: Eukaryotic elongation factor 2
EGFR: Epidermal growth factor receptor
ENOL‐1: Enolase 1
FC: Fold change
FDR: False discovery rate
GEO: Gene Expression Omnibus
GO: Gene Ontology
GOT: Glutamate‐oxaloacetate transaminase
GTP: Guanosine triphosphate
HIF1A: Hypoxia inducible factor 1 subunit alpha
HPHD‐1: Hydroxyacid‐oxoacid transhydrogenase mitochondrial
HSP: Heat shock protein
ImaGEO: Integrative Meta‐Analysis of GEO
ISR: Integrated stress response
KEGG: Kyoto Encyclopedia of Genes and Genomes
KRAS: Kirsten rat sarcoma
MCODE: Molecular complex detection
MDH: Malate dehydrogenase
Met: Methionine
MF: Molecular function
MRP: Mitochondrial ribosomal protein
MYO: Myosin
NCBI: National Center for Biotechnology Information
NDUFAB1: Ubiquinone Oxidoreductase Subunit AB1
PCA: Principal component analysis
PPI: Protein–protein network
PRKACB: Protein kinase CAMP‐activated catalytic subunit beta
PYP‐1: Inorganic pyrophosphatase 1
qPCR: Real‐time polymerase chain reaction
Q‐ToF: Quadrupole time‐of‐flight
RNA: Ribonucleic acid
RPL: Ribosomal protein L
RRID: Research resource identifier
SIP‐1: Stress‐induced protein 1
T08H10.1: NADP‐dependent oxidoreductase domain‐containing protein
TP53: Tumor protein P53
TPI‐1: Triosephosphate isomerase
UBC: Ubiquitin C
UNC‐54: UNCoordinated‐54

## 1 Introduction

Alzheimer's disease (AD) is the predominant form of dementia, posing urgent and emerging challenges worldwide (Zhang, Zhang, et al. 2024). Scaling population aging is one of the challenges as the number of individuals with AD increases as the population ages, which is a global trend (Alzheimer's 2023). In 2019, the World Health Organization estimated that 52.2 million individuals were living with dementia (Brain Health (BRH) 2022). Among individuals aged 65 and older, 1 in 10 has AD (Liu, Tan, et al. 2024). By 2030, the number of people with dementia will increase to 78 million, with global economic surpluses of US$2.8 trillion in managing individuals (Brain Health (BRH) 2022).

AD is characterized by the deposition of β‐amyloid (Aβ) and intracellular accumulations of hyperphosphorylated Tau, forming senile plaques (Liu, Zhang, et al. 2024). Cognitive disorders related to memory deterioration, aphasia, declines in visuospatial skills, and changes in behavior and personality are some of the clinical markers of AD (McKhann et al. 1984; Mirzaei and Adeli 2022). The pathophysiology in neuronal tissue comprises increased Aβ deposits generating mitochondrial damage, oxidative stress, synaptic dysfunction, and decreased aerobic respiration, among others (Gao et al. 2020; Fani et al. 2021; Hong et al. 2016; Kugler et al. 2024). Also, neuronal loss and synaptic dysfunction are the primary hallmarks of AD (Reiss et al. 2023). However, treatments are currently ineffective in terms of the genesis of disease development (Lopez Lopez et al. 2019).

Experimental models for AD can be broadly categorized based on pathological and pathophysiological factors into spontaneous, interventional, and genetically modified models (Chen and Zhang 2022). Vertebrate models such as rats, mice, canines, zebrafish, and non‐human primates have been extensively used to study AD pathology and potential therapies (Chen and Zhang 2022; Sharma et al. 2017). These models have provided valuable insights due to their physiological similarities to humans, including complex brain structures and cognitive behaviors. However, they come with significant limitations such as ethical concerns, high maintenance costs, long life spans leading to prolonged study durations, and the relatively small number of animals used, which can limit statistical power (Sharma et al. 2017; Vitek et al. 2020).

In contrast, invertebrate models like *Caenorhabditis elegans* ( *C. elegans* ) offer unique advantages despite their physiological distance from humans (Drummond and Wisniewski 2017). They enable the rapid generation of knockout or overexpression lines, facilitate large‐scale toxicity and recovery assays, and support survival studies in a high‐throughput and cost‐effective manner (Lublin and Link 2013; Griffin et al. 2017). Although the simplicity of *C. elegans* limits direct translation of some complex neural aspects of AD, its genetic tractability and scalability make it an indispensable tool for dissecting molecular mechanisms and screening therapeutic candidates. Thus, combining vertebrate and invertebrate models provides complementary perspectives that are crucial for advancing AD research.

*C. elegans* is a free‐living nematode applied in studies about cell and developmental biology (da Silva et al. 2024). The short lifespan, transparency of the body, self‐fertilizing hermaphroditism, and genetic similarity with humans are also essential characteristics (Brenner 1974). *C. elegans* has the post‐embryonic larval stages of L1–L4, where they become adult animals (Baugh 2013). Adults, which measure approximately 1 mm, present an average lifespan of approximately 18–20 days when cultured at 20°C (Klass 1977). Furthermore, *C. elegans* has genetic similarities with humans, where around 38% of the genes have human orthologs, and the proteome has 83% homology with human genes ( *C. elegans* Sequencing Consortium 1998; Shaye and Greenwald 2011; Lai et al. 2000). The advancement of biotechnology has taken the model to a new stage, enabled by the potential for genetic manipulation through transgenics. This means it is possible to create animals that contain genetic characteristics of human diseases, such as AD.

The *C. elegans dvIs2* [pCL12(unc‐54/human Abeta peptide 1–42 minigene) + rol‐6(su1006)] (CL2006) transgenic model constitutively expresses the Aβ peptide in body muscles, containing an *unc‐54* promoter region encoding 42 amino acids of Aβ (Aβ_1–42_) (Link 1995). Furthermore, the Roller phenotype is a behavioral marker of Aβ deposits present in the animal, guaranteed by the insertion of the collagen mutant gene ([rol‐6(su1006)]) that produces a dominant and distinctive behavior from other non‐transgenic animals (Link 1995). In *C. elegans* , Aβ deposits and oligomers cause paralysis and toxicity (Alvarez et al. 2022). Aβ alters proteostasis in *dvIs2* (CL2006) animals (Kikis 2016), in addition to modifying the gene expression profile (Mukherjee et al. 2017) and increasing oxidative stress (Wan et al. 2011).

In the present study, we characterized the proteome of the AD model in *C. elegans* . Furthermore, we analyzed datasets of human individuals with AD and identified by gene orthology the sharing of common orthologous genes for AD in *C. elegans* . Then, the *C. elegans* orthologs were compared to the proteins identified in proteomics. Furthermore, we identified key regulators in protein–protein interaction (PPI) networks and their topological properties in humans and compared them with the key regulators and topological properties of *C. elegans* . Proteome data, containing orthologous proteins shared among organisms for AD, were also used in Gene Ontology (GO) analyses and to identify the involved pathways. The trade‐off between sharing genes and proteins between individuals with AD and the AD model in *C. elegans* can make a critical contribution to the use of the model. To our knowledge, this is the first study on the omics characterization of the *dvIs2* (CL2006) AD model in *C. elegans* .

## 2 Materials and Methods

### 2.1 Proteomics Analysis

#### 2.1.1 *C. elegans* Strains and Maintenance

Worms were cultivated in Petri dishes containing nematode growth medium (cat. no. K25‐1800) (NGM) composed of 3 g NaCl (cat. no. 7647‐14‐5), 2.5 g peptone (cat. no. 102314468), 17 g agar, and 975 mL autoclaved H_2_O; further added 1 mL 1 mol L^−1^ CaCl_2_ (cat. no. C2013.01.AG), 1 mL 5 mg mL^−1^ cholesterol in ethanol (cat. no. C8503), 1 mL 1 mol L^−1^ MgSO_4_ (cat. no. 10034‐99‐8), and 25 mL 1 mol L^−1^ KPO_4_ buffer (cas. no. F2002.01.AG) and fed with *Escherichia coli* OP50 (RRID:WB-STRAIN:WBStrain00041969) under controlled temperature (20°C) to N2 wild‐type strain (RRID:WB-STRAIN:WBStrain00000001) and 15°C to *dvIs2* [pCL12(unc‐54/human Abeta peptide 1‐42 minigene) + rol‐6(su1006)] (CL2006) strain (RRID:WB-STRAIN:WBStrain00005094). The humidity was around > 95% (Stiernagle 2006). The population was synchronized to obtain all worms at the same larval stage. For this, we used a bleaching solution (NaOH 1 M (cat. no. S5881), NaClO 1% (cat. no. 239305), and distilled H_2_O) to break the cuticle of pregnant hermaphrodites and release the eggs. Eggs were kept in the M9 buffer (3 g L^−1^ KH_2_PO4 (cat. no. F1046.01.AG), 6 g L^−1^ Na_2_HPO_4_ (cat. no. 7558‐79‐4), g L^−1^ NaCl, 1 mM MgSO_4_ (cat. no. 10034‐99‐8)) until hatching and reached the L1 larval stage at 20°C. The wild‐type N2 strain (Bristol) and AD model (CL2006) of *C. elegans* and *E. coli* were obtained from the *Caenorhabditis* Genetics Center (CGC, Minnesota, USA).

#### 2.1.2 Protein Sample Extraction, Quantification, Clarification, and Digestion

Approximately ~15,000 worms of each group were used. At the L4 larval stage, the animals were washed six times with type II ultrapure water to completely remove *E. coli* . After washing, the worms were subjected to protein extraction (Figure 2H). The number of animals was based on experimental designs reported in previous studies involving *C. elegans* proteomics, where similar sample sizes yielded statistically robust and biologically relevant results (Parmar et al. 2021). The extraction was performed by manual maceration using the Sample Grinding KitTM (Cytiva, Marlborough, MA, cat. no. 80‐6483‐37) following the manufacturer's instructions and using a lysis buffer (7 M urea (cat. no. U5128), 2 M thiourea (cat. no. T7875), 4% 3‐[(3cholamidopropyl)dimethylammonio]‐1‐propanesulfonate (cat. no. C3023), 40 mM DTT (cat. no. D0632), 0.5% Immobilized pH Gradient Buffer). The samples were centrifuged at 10,000 g for 5 min, and the supernatant was recovered. During extraction, a protease inhibitor cocktail (Promega, Madison, Wisconsin, USA, cat. no. G6521) was added using the protein ratio of 1:50 w/w to the mixture.

Following the extraction, the protein concentration was determined using 2‐D Quant Kit (Cytiva, Marlborough, MA, cat. no. 80‐6483‐56) following the manufacturer's instructions. Afterwards, 100 μg of protein from each group was submitted to a 2‐D Clean‐Up Kit (Cytiva, Marlborough, MA, cat. no. 80‐6483‐56) protocol to remove any interfering components. Approximately 100 μg of protein from each group was resuspended in 8 M urea (cat. no. U5128). The proteins were reduced by adding 100 mM dithiothreitol (cat. no. D0632) at 30°C for 30 min and alkylated with 300 mM iodoacetamide for 30 min. Afterwards, 50 mM NH_4_HCO_3_ (cat. no. 09830) was added to the solution. For protein digestion, sequencing‐grade modified trypsin (Madison, Wisconsin, USA, cat. no. T6567) was added at a protein/protein ratio of 1:50 w/w, and the samples were incubated at 37°C for 18 h. The tryptic peptides were centrifuged at 11,000 g for 10 min at 4°C, and the supernatant was transferred to a protein LoBind tubing from Eppendorf (Hamburg, Germany, cat. no. 56251) and concentrated at 30°C on a Speed Vacuum concentrator 5301 Eppendorf (Hamburg, Germany) (de Farias et al. 2024).

#### 2.1.3 Liquid Chromatography‐Mass Spectrometry/Mass Spectrometry (LC–MS/MS) Analysis

After digestion, the samples of tryptic peptides were recuperated in 0.1% formic acid (cat. no. 27001) so that the final concentrations of 1 μg μL^−1^ for protein were reached and transferred to a total recovery vial. The tryptic peptides were separated using an M‐Class ACQUITY UPLC system (Waters Corporation, Milford, MA). This system was equipped with a Single Pump Trap with a column: nanoease M/Z symmetry C18 100 A, 5 μm, 180 μm × 20 mm trap column, operating at 0.3 μL min^−1^ coupled to an analytical nanoease M/Z HSS C18 T3, 100 A, 1.8 μm, 75 μm × 250 mm column operating at 0.3 μL min^−1^ and a temperature of 40°C. The mobile phase A consisted of water containing 0.1% (V/V) formic acid, while the mobile phase B consisted of acetonitrile containing 0.1% (V/V) formic acid. Peptide elution was performed for 133 min and a loading time of 10 min using the following gradient: (I) 0–8 min, 95% of A and 5% of B; (II) 8–98 min, 60% of A and 40% of B; (III) 98–103 min, 15% of A and 85% of B; (IV) 103–108 min, 15% of A and 85% of B; (V) 108–109 min, 95% of A and 5% of B; (VI) 109–133 min, 95% of A and 5% of B (starting conditions).

The system was coupled to a Synapt XS quadrupole (Q‐ToF) mass spectrometer with T‐Wave Ion Mobility (Waters Corporation, Wilmslow, UK) operating at a mass resolution of 30,000 full width at half maximum. The electrospray low flow probe capillary voltage was 3 kV, sampling cone 40 V, source offset 30 V, source temperature 100°C, and cone gas 50 L min^−1^. The time‐of‐flight mass analyzer was externally calibrated with a NaCsI mixture from *m*/*z* 50 to 2000. A lock mass reference signal of GluFibrinopeptide B (*m*/*z* 785.8426) was sampled every 30 s. Data were acquired in triplicate using the Ultra‐Definition Mass Spectrometry in positive ion mode.

#### 2.1.4 Data Processing, Protein Identification, and Quantification

Protein identification was performed on LC–MS/MS data using Progenesis QI v 4.7 software (Nonlinear Dynamics) with a revised and non‐revised protein database that includes the *C. elegans* proteome (UniProt_download on 09/12/2024) (Bateman et al. 2023). The parameters for spectra processing and database search were loss of trypsin cleavage, max protein mass of 750 kDa, fixed modification of carbamidomethyl to Cysteine (Cys), and variable modification of oxidation to methionine (Met). The search thresholds used were (2) minimum fragment ion matches per peptide, (5) minimum fragment ion matches per protein, (2) minimum fragment ion match peptides per protein, and (1) false discovery rate (FDR) for protein identification. Relative quantification was performed using the three most abundant peptides for all identified proteins (Hi3 label‐free method). Only proteins with frequency scores and confidence intervals greater than 99% were considered acceptable according to the algorithm in the database searches.

#### 2.1.5 Validation of Transgenic Animals by RT‐qPCR

RT‐qPCR was performed using the ViiA 7 Real‐Time PCR System (Applied Biosystems) to confirm the transgene expression in the strain *dvls2* (CL2006). After the animals reached the L4 larval stage (~44 h), six washes with type II ultrapure water were performed to remove the bacteria. Then, ~2,000 worms were used for RNA isolation. No sample size calculation was performed; however, the sample size was determined by previous studies (Koyunku et al. 2021). Primers were provided by Exxtend, described as follows: Aβ F: CCGACATGACTCAGGATATGAAGT, and R: CACCATGAGTCCAATGATTGCA (Yang et al. 2017). Actin F: TCGGTATGGGACAGAAGGAC, and R: CATCCCAGTTGGTGACGATA. cDNA was constructed using the High‐Capacity cDNA Reverse Transcription Kit (Thermo Fisher Scientific, cat. no. 4368814). The qPCR reaction was carried out using SYBR Green Master Mix (Promega, USA, cat. no.). The cycling conditions were as follows: an initial denaturation at 95°C for 2 min, followed by 45 cycles of 95°C for 3 s and 60°C for 30 s. A melting curve analysis was performed to confirm the specificity of amplification. Gene expression levels were analyzed using the ΔCt method and plotted with GraphPad Prism 8.0 (GraphPad Software, San Diego, CA, USA).

#### 2.1.6 Bioinformatics Analysis of Proteomics Results

The Progenesis QI v4.7 software was used to identify, normalize, and extract protein groups. Normalization is done by scaling abundances based on the median of the logarithmic ratios between each sample and a reference sample. After filtering outliers, a scaling factor is calculated to adjust abundances, thereby minimizing technical variations (Ferguson et al. 2005). The normalized abundances were transformed into log^2^; then, a t‐test was applied, using *p* ≤ 0.05 as the significance threshold. The Benjamini‐Hochberg test was used to minimize the number of false positives and preserve sensitivity. The adjusted *p*‐value was used for protein analysis, and the statistical details are available in Table S5. The Omicscope 1.4.2 algorithm (Python 3.8) was used for differentially expressed proteins (DEPs) identification (Reis‐de‐Oliveira et al. 2024). The in‐house scripts with Python 3.12 were used to analyze some data and are available on GitHub (https://github.com/Conradoou/Proteomics.git), and no test for outliers was conducted. Principal component analysis (PCA) was conducted to observe the separation of the samples, and then a volcano plot was used to separate up‐ and downregulated proteins. To improve symmetry and approximate the normal distribution, the data were transformed into the log^2^ of the fold change (FC) and the ‐log^10^ of the adjusted *p*‐value to generate a volcano plot with FC cutoffs of ≤ −1.2 and ≥ 1.2 and a *p*‐value of 0.05. Additionally, we calculated the average coefficient of variation to assess the reproducibility between replicates (Xia et al. 2018; Brenes 2024). The coefficient of variation was calculated using normalized abundance with the following search Equation (1):

(1) $$\frac{\text{Standard deviation}}{\text{Normalized abundance mean}}\times 100$$

Based on the Gene Ontology database, the biological process (BP), cellular component (CC), molecular function (MF), KEGG, and Reactome were performed using the DAVID (Sherman et al. 2022). The DEPs above the FC cutoff were used in the ontology analysis. The statistical significance was set at a *p*‐value < 0.05, and the top 10 results were plotted.

### 2.2 Conserved Genes and Pathways in *C. elegans* Analysis

#### 2.2.1 Identification of AD Genes in Humans

The datasets were obtained from the Gene Expression Omnibus (GEO) from the National Center for Biotechnology Information (NCBI) (https://www.ncbi.nlm.nih.gov/geo/). Three microarray datasets, GSE48350, GSE5281, and GSE36980, were selected for the study. General information about the GEO datasets used is in Table 1. The meta‐analysis was performed with the Integrative Meta‐Analysis of GEO Data (ImaGEO), a web tool that implements a complete and comprehensive meta‐analysis workflow starting from GEO dataset identifiers (Toro‐Domínguez et al. 2019). The parameters for meta‐analysis were the *p*‐value and Fisher's exact test, with the adjusted *p*‐value threshold ≤ 0.05. Log FC was used to analyze differentially expressed genes (DEGs) with ≤ −0.15 and ≥ 0.15 cutoffs. DEGs above the FC cutoff were used in the screening of orthologous genes in *C. elegans* .

**TABLE 1:** Information about the GEO datasets used in the study.

Datasets	Control	Cases	Platform
GSE48350	173	80	GPL570
GSE5281	74	87	GPL570
GSE36980	47	33	GPL6244

#### 2.2.2 Construction of PPI Network for Human Alzheimer's Disease and Identification of Core Genes

The PPI network was constructed to understand the regulatory function of human DEGs for AD. STRING was used to build the network (Szklarczyk et al. 2023). STRING physical integration data was imported into Cytoscape 3.10.1 (Shannon et al. 2003). Finally, a PPI network of DEGs was constructed after removing isolated nodes. The Cytohubba plugin was used to analyze the top link properties of the network (Chin et al. 2014).

The influential nodes in the PPI network are considered the hubs. For the identification of key regulators, the degree and centrality measurements (closeness (*C*_*C*_) and betweenness (*C*_*B*_) centrality) were used to identify them. Next, a Venn diagram was generated to identify central genes between degree, *C*_*C*_, and *C*_*B*_.

#### 2.2.3 Identification of Alzheimer's Disease‐Related Orthologous Genes in *C. elegans*

These analyses were adapted from Ray et al. (2024). The human genes found in the AD datasets were prospected for orthologous genes in *C. elegans* . The genes found were taken to Ortholist 2, a server with a compilation of *C. elegans* –human orthologs obtained by meta‐analysis in 2018 (Kim et al. 2018). Orthologs in *C. elegans* were generated, and comparative analysis with Python 3.10 was used to identify the presence of the same genes in the proteomic data obtained previously from the *C. elegans* AD model. The common genes in proteomics and AD orthologs were obtained and used in subsequent analyses (Ray et al. 2024).

#### 2.2.4 *C. elegans* PPI Network Ortholog Construction and Analysis

Animal models for human diseases are used to understand where genes and proteins act in their regulation. The acquisition of human genes and proteins with that of *C. elegans* may suggest similar functionalities and roles in AD, with possible conserved interactions (Kotlyar et al. 2016). Therefore, we constructed the *C. elegans* PPI network using the proteomic results with the DEPs. We use the Omics Visualizer plugin in Cytoscape 3.10.1 (Shannon et al. 2003). The network was constructed with the STRING database (https://string‐db.org/) (Szklarczyk et al. 2023). After removing isolated nodes, a network of 356 nodes and 3003 edges was constructed.

The degree, *C*_*C*_, and *C*_*B*_ measurements of each node were analyzed using the CytoHubba plugin in Cytoscape 3.10.1 to evaluate the relevance of some central genes within the interaction network (Chin et al. 2014). Furthermore, a Venn diagram was created to identify the central proteins between degree, *C*_*C*_, and *C*_*B*_. The overlapping between groups was used to compare the list of AD orthologous genes in *C. elegans* .

#### 2.2.5 Identification of Key Regulators in the *C. elegans* Orthologous Network

After identifying the main human genes and *C. elegans* proteins based on degree, C_C_, and *C*_*B*_, a search was conducted for key regulators common to both organisms and their networks. Key regulators with significant roles in orchestrating PPI networks were among the hubs identified in previous sessions. Therefore, a group of nodes with high degree and centrality measures using Cytoscape 3.10.1 was used to identify key regulators. Hubs functioning as the backbone and system‐level structure have been tracked and found to be significant regulators.

#### 2.2.6 Analysis of Modules in the *C. elegans* Network

**FIGURE 1:**
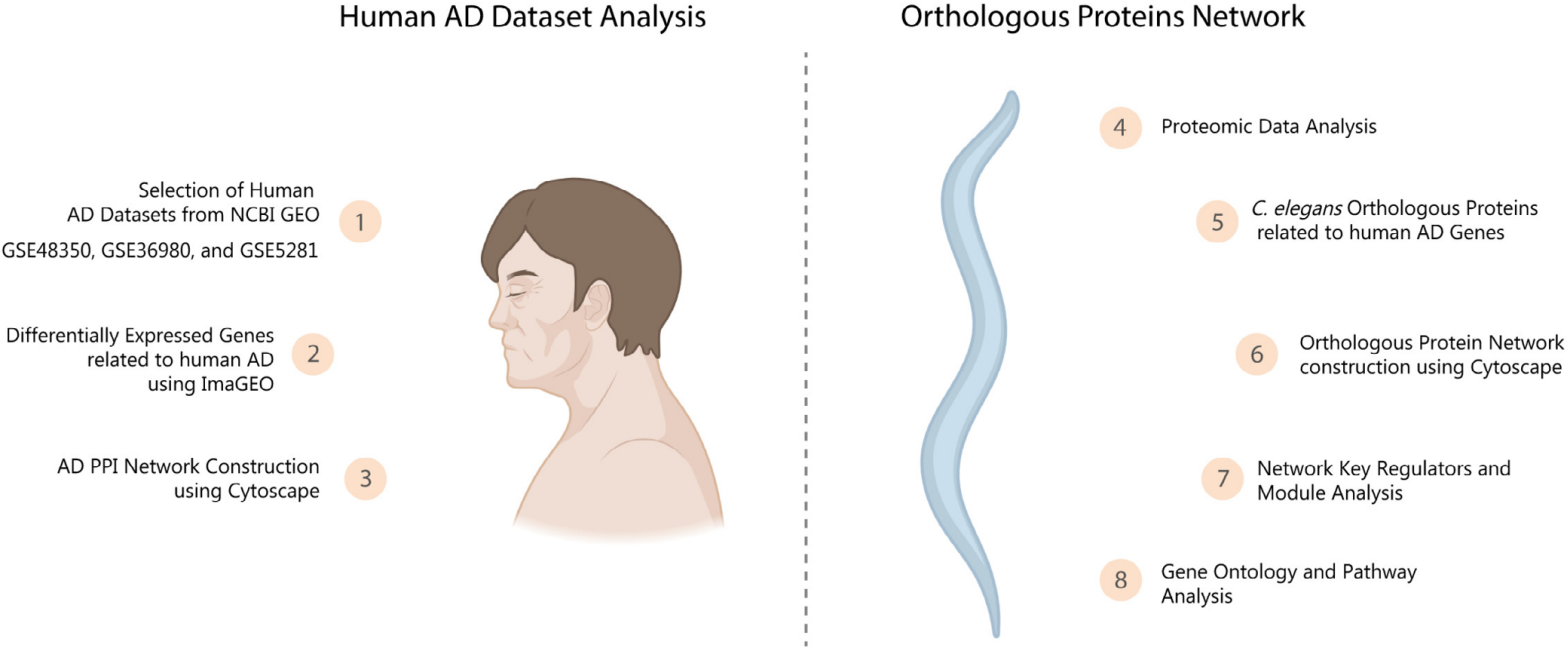
Methodological outline of the study. 1. Selection of human microarray datasets from AD patients; 2. Meta‐analysis of datasets to obtain differentially expressed genes among the datasets using ImaGEO; 3. Construction of the PPI network of AD genes in humans, in addition to characterization of the network topology and identification of *hubs* and key regulators; 4. Analysis of proteomic data from *C. elegans dvls2* (CL2006); 5. Identification of orthologous genes/proteins to human AD genes using Ortholist. After this, a comparative analysis of the orthologous genes with the DEPs identified in proteomics was performed, generating a list of proteins orthologous to the human AD genes; 6. Construction of the PPI network of AD orthologs in *C. elegans* and *hubs* identification; 7. Identification of key regulators in the PPI network and analysis of the main network modules; 8. Gene ontology analysis and pathway analyses by KEGG and Reactome.

Modules represent proteins that tend to interact with each other more frequently than with proteins outside the module (Guimarães et al. 2018). The molecular complex detection (MCODE) plugin version 2.0.3 in Cytoscape 3.10.1 was used to build the modules, generating clusters (regions with a high density of connections) (Bader and Hogue 2003). The MCODE parameters were “Degree cutoff = 2”, “node score cutoff = 0.2”, “k‐score = 2”, and “max. Depth = 100”. Modules with a score ≥ 5 were considered significant and filtered from the PPI network. Figure 1 shows the general methodological scheme used in the study.

## 3 Results and Discussion

AD is the most predominant form of dementia, with significant impacts on healthspan by affecting various cognitive activities. The etiology of the disease is still not well understood, as it is complex, resulting from genetic and environmental changes (Zhang, Zhang, et al. 2024). Applying animal models to study AD provides valuable insights into its pathological characteristics, such as toxicity induced by Aβ and Tau peptides, neurodegeneration, and behavioral impacts (Zhong et al. 2024). Our study uses proteomics and bioinformatics approaches to characterize the animal model of AD in *C. elegans* . *C. elegans* has been used in several studies that investigate potential molecules against the aggregation of the Aβ peptide, focusing on the behavioral, genetic, and toxicological aspects of the disease (McColl et al. 2009; Shanmuganathan et al. 2019). Furthermore, we identified genes for human AD that are orthologs of genes in the *C. elegans* AD model.

The *dvls2* (CL2006) strain is widely used in the prospecting of new molecules with antiaggregant potential. Some researchers evaluate the capacity of new potential drugs to extend the life of animals, increase resistance to stress, decrease body paralysis, and enhance chemotactic responses (Yang et al. 2017). In addition, numerous proteins have high similarity to human proteins, such as acetylcholinesterase, mitochondrial enzymes, and presenilin, among others, which play roles in the pathophysiology of AD (Paul et al. 2020). We evaluated the presence of the transgene by RT‐qPCR and identified that *dvIs2* (CL2006) animals were expressing the Aβ gene before starting our experiments (Figure S1).

**FIGURE 2:**
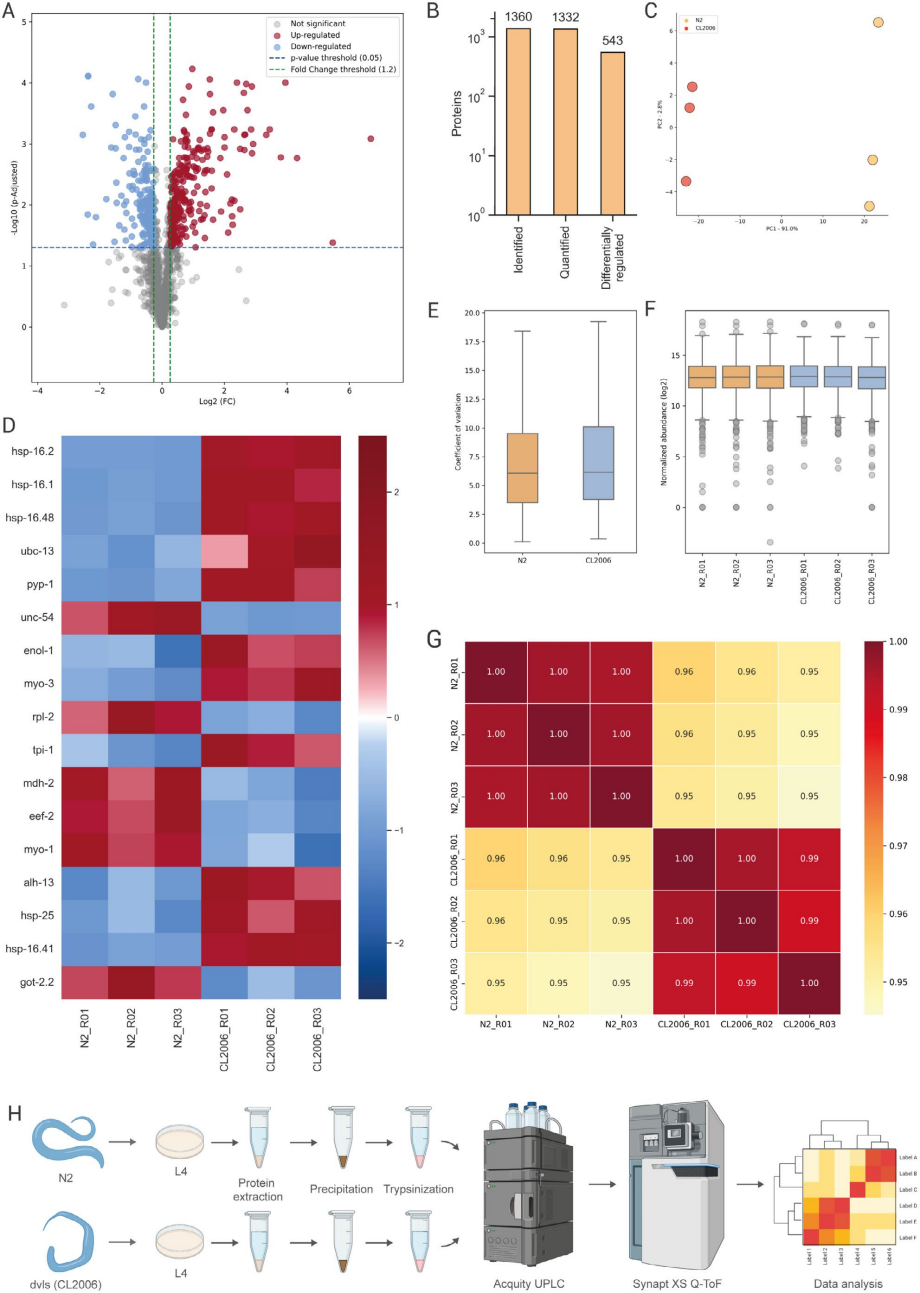
Proteomic analysis of the *dvls2* (CL2006) *C. elegans* strain. (A) Volcano plot of the proteomic profile of *dvls2* (CL2006) animals compared to wild‐type, with Log^2^ FC of ≥ 1.2 and ≤ −1.2 and *p* ≤ 0.05. (B) Data of identified, quantified, and differentially regulated proteins. (C) Principal components analysis of data. (D) Heatmap of differentially expressed proteins with repercussions on AD. (E) Coefficient of variation of proteomics replicates. (F) Data normalization. (G) The correlation coefficient between study replicates. (H) Simplified schematic drawing of the extraction process and obtaining of peptides for ingestion, identification, and analysis.

The volcano plot was generated, and the DEPs, using the cutoff limit of FC ≥ 1.2 and ≤ −1.2 and *p* ≤ 0.05, were identified, where 250 proteins were upregulated and 220 proteins were downregulated (Figure 2A). We identified and quantified 1360 and 1332 proteins, respectively. However, only 543 proteins were differentially regulated (Figure 2B).

The PCA showed a distinction between the groups (Figure 2C). The PCA of *dvIs2* (CL2006) showed a PC1 of 91% and a PC2 of 2.7%. Data normalization was performed to ensure more accurate comparisons and presented concise mean abundances and error ranges across all conditions and replicates (Figure 2F). Our proteome analysis revealed high data reproducibility and a wide range of identified proteins, corroborated by the dynamic range (Figure S2). Additionally, the average coefficient of variation of the detected proteins was 8.6% for wild‐type animals (N2 worms) and 9.5% for the *dvIs2* (CL2006). Figure 2E shows the coefficient of variation (the outliers were removed for better visualization). The low coefficient of variation (≤ 30%) observed across replicates reflects the high reproducibility of the proteomic measurements, consistent with values reported in previous proteomic studies involving *C. elegans* (Xia et al. 2018). The high coefficient of variation of some proteins may be due to discrepancies in sampling times between different runs and/or difficulties in detecting low abundance using MS/MS. To visually explore patterns of expression and variability across samples, a heatmap was generated, providing a comprehensive overview of differentially expressed proteins (Figure 2D).

Pearson correlation analysis was performed to identify pairwise correlations between samples and identify outliers, technical variations, reproducibility, and normalization issues. All experimental groups showed high correlations between replicates of the same group (Figure 2G).

**FIGURE 3:**
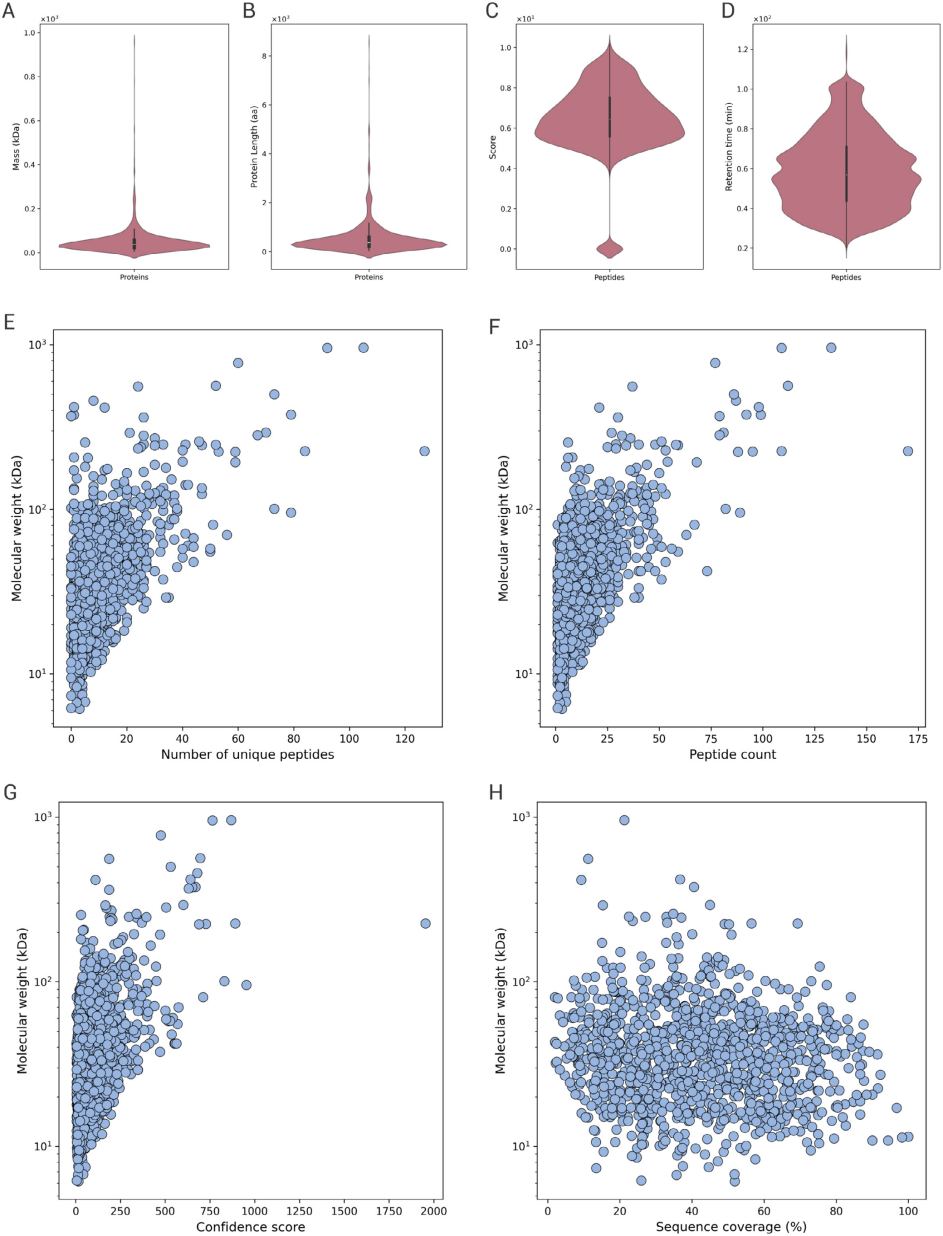
Violin plot representation of the relationship between identification parameters. (A) Mass in kDa of the identified proteins. (B) Protein size. (C) Fragmentation score. (D) Peptide retention time. (E) Relationship between molecular weight and number of unique peptides. (F) Relationship between molecular weight and peptide count. (G) Relationship between molecular weight and confidence score. (H) Sequence coverage of the identified peptides in relation to the total constituent amino acids of the target protein.

Protein data were processed and evaluated to obtain proteomic profiles. The molecular mass distribution of the identified proteins revealed an asymmetric trend with a strong concentration of proteins in lower mass bands (Figure 3A). Besides, most proteins were concentrated in lengths below 1 × 10^3^ amino acids (aa), suggesting the predominance of moderate‐sized proteins (Figure 3B). The peptide identification score revealed confidence levels considered acceptable in proteomic studies, where the symmetrical shape of data distribution suggests homogeneity in peptide identification (Figure 3C). In addition, the peptide retention time distribution showed an identification profile consistent with expected chromatographic separation patterns, suggesting good efficiency (Figure 3D).

Furthermore, corresponding relationships between molecular weight and the number of unique peptides (Figure 3E), peptide count (Figure 3F), confidence score (Figure 3G), and sequence coverage (Figure 3H) were identified.

Three datasets of AD in humans were obtained to gain insights into AD‐related genes and pathways in *C. elegans* . Details of the datasets used are presented in Table 1. The selected datasets were analyzed in ImaGEO through a meta‐analysis. DEGs screening was carried out with a threshold of *p‐*value set as ≤ 0.05, where all datasets passed quality control (Table S1). The meta‐analysis detected 4019 significantly altered genes used in the analyses (Table S3). Finally, according to the FC cutoff, 397 upregulated DEGs and 767 downregulated DEGs were associated with AD. The DEGs obtained were used to map orthologous genes in *C. elegans* .

Genetic sequencing of *C. elegans* in 1998 showed that ~38% of the animal's genes have human orthologs (Kim et al. 2018). Moreover, comparative proteomics revealed that 83% of the *C. elegans* proteome has homology with human genes (Ray et al. 2024). To identify and understand the possible human orthologous genes in *C. elegans* , the identified human DEGs were analyzed in Ortholist 2, revealing 1325 orthologous genes (Table S4). The orthologous genes were used for comparative analysis with the proteome (based on gene ID) of the *dvIs2* (CL2006), followed by the construction of a PPI network. In the comparative study of *C. elegans* *dvIs2* (CL2006) orthologous genes with proteomics data, we identified 63 common proteins, 29 upregulated and 24 downregulated DEPs, according to the FC cutoff (Table S2). The comparative analysis of orthologous human DEGs and *C. elegans* *dvIs2* (CL2006) DEPs helps in understanding possible targets related to AD, which guarantees new insights into the animal model.

**FIGURE 4:**
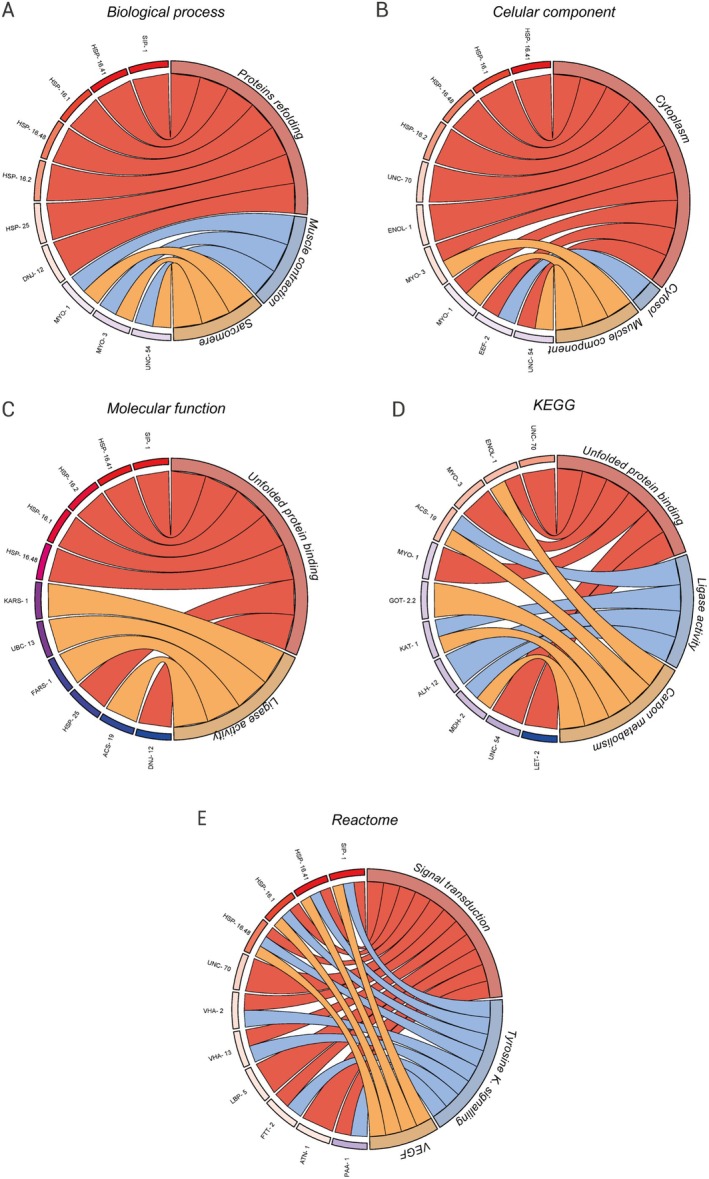
Circle plot of signaling pathways enrichment analysis. (A) Biological processes. (B) Cellular component. (C) Molecular function. (D) KEGG and (E) Reactome. The red color next to the *C. elegans* protein names represents upregulation, while the blue color represents downregulated proteins.

The functional enrichment of the 29 upregulated and 24 downregulated DEPs of the *dvIs2* (CL2006) strain was performed with DAVID. In BP, gene annotations were made related to protein refolding (GO:0042026) (*p* < 0.0001), muscle contraction (GO:0006936) (*p* < 0.01), and sarcomere organization (GO:0045214) (*p* < 0.01) (Figures 4A and S3A). In CC, we observed that the enriched genes predominantly constitute cytoplasm/cytosol (GO:0005737; GO:0005829) (*p* < 0.0001) and muscle components (Figures 4B and S3B). MF was enriched for unfolded protein binding (GO:0051082) (*p* < 0.0001) and, in addition to Ligase activity (GO:0016874) (*p* < 0.01) (Figures 4C and S3C). Furthermore, when analyzing the KEGG pathways, the proteins were enriched for cytoskeleton in muscle cells (cel04820) (*p* < 0.001), pyruvate metabolism (cel00620) (*p* < 0.01), and carbon metabolism (cel01200) (*p* < 0.05) (Figures 4D and S3D), and annotations in signal transduction (*p* < 0.05) were obtained from reactome, in addition to signaling by tyrosine kinase receptor (*p* < 0.001), and signaling by VEGF pathways (Figures 4E and S3E).

Cells have highly controlled mechanisms of protein folding and assembly with the ability to maintain protein folding and organize three‐dimensional structures and specific domains (Díaz‐Villanueva et al. 2015). In AD, the expression and aggregation of Aβ peptides can generate toxicity since the aggregation produces different arrangements of oligomers, producing different toxic species by mixing distinct molecular populations (Stefani and Dobson 2003). In the AD model in *C. elegans dvIs2* (CL2006), the aggregation of the Aβ peptide triggers proteotoxic processes through the accumulation and formation of Aβ fibrils (Alvarez et al. 2022).

**FIGURE 5:**
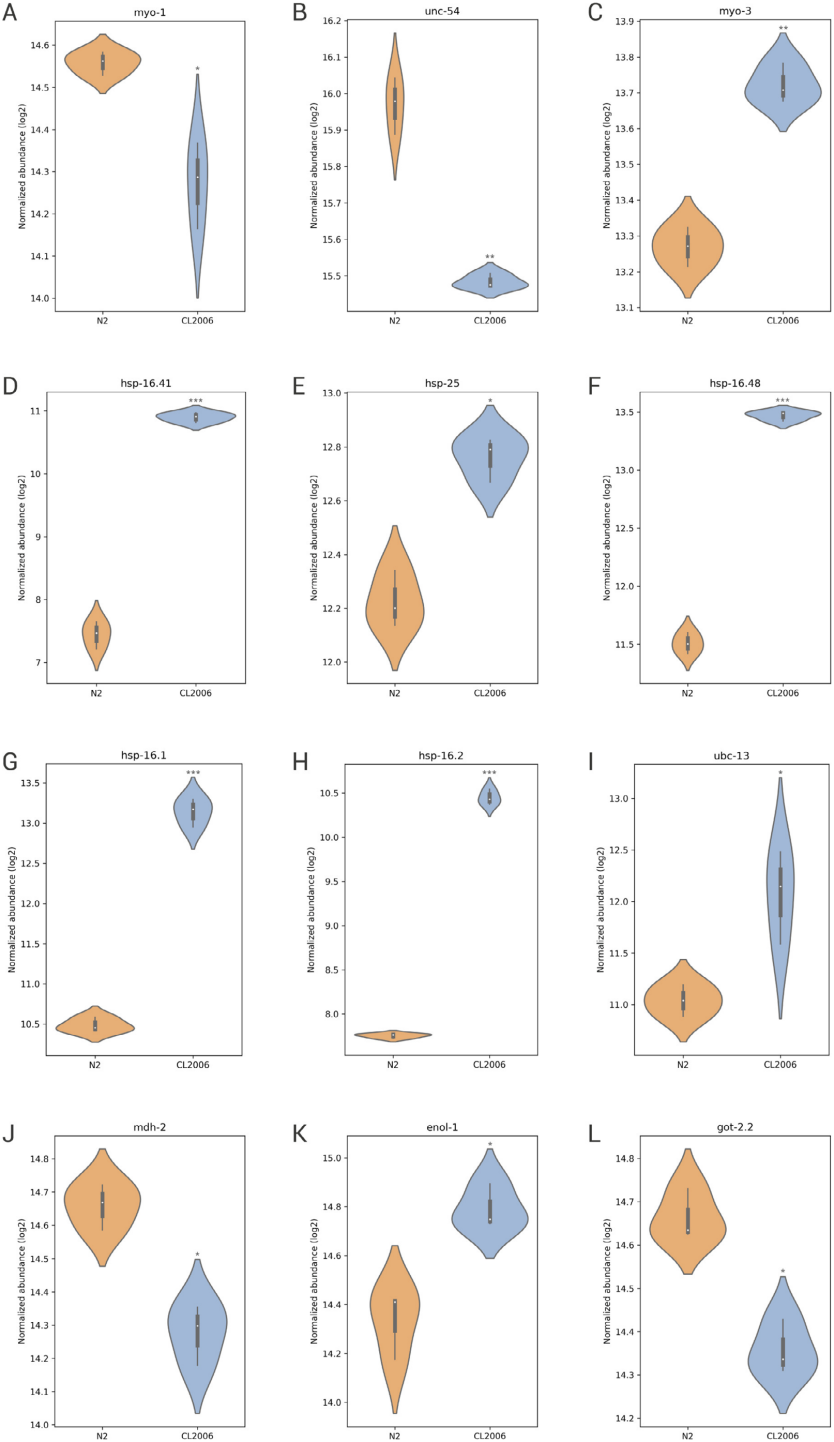
Violin plot visualization of protein expression in N2 wild‐type and *dvls2* (CL2006) by normalized abundance. (A) Myosin‐1 (MYO‐1). (B) UNCoordinated‐54 (UNC‐54). (C) Myosin‐3 (MYO‐3). (D) Heat shock protein 16.41 (HSP‐16.41). (E) Heat shock protein 25 (HSP‐25). (F) Heat shock protein 16.48 (HSP‐16.48). (G) Heat shock protein 16.1 (HSP‐16.1). (H) Heat shock protein 16.2 (HSP‐16.2). (I) Ubiquitin C (UBC‐13). (J) Malate dehydrogenase (MDH‐2). (K) Enolase‐1 (ENOL‐1). (L) Glutamate‐oxaloacetate (GOT‐2.2). Data are represented as distribution plots, with median and quartiles indicated. Statistical significance was determined by a t‐test followed by the Benjamini‐Hochberg procedure (FDR). *p < 0.05, **p < 0.01, ***p < 0.001.

We hypothesize that proteins related to the refolding of other proteins were mobilized to repair the formed Aβ fibrils. Furthermore, we propose that proteins related to muscle activity, such as muscle contraction and sarcomere organization, are enriched since *dvIs2* (CL2006) animals have muscle dysfunctions due to the expression of the Aβ peptide (Figure 4A,B) (Link 1995). The myosin proteins MYO‐1 (*p* < 0.05) (Figure 5A) and UNC‐54 (*p* < 0.01) (Figure 5B) were downregulated, while MYO‐3 was upregulated (*p* < 0.01) (Figure 5C); such proteins play a role in the animal's muscular physiological processes. Additionally, changes in myosin are observed in individuals with AD, as they have morphological changes in neurons due to myosin dysfunctions, altering axonal morphology (Fu et al. 2023).

Some proteins were enriched for interacting with unfolded proteins (Figure 4A,C). The chaperone proteins HSP‐16.41 (*p* < 0.001) (Figure 5D), HSP‐25 (*p* < 0.05) (Figure 5E), HSP‐16.48 (*p* < 0.001) (Figure 5F), HSP‐16.1 (*p* < 0.001) (Figure 5G), and HSP‐16.2 (*p* < 0.001) (Figure 5H) were upregulated. Previous studies have observed that chaperones bind intracellularly to soluble Aβ monomers and oligomers in *C. elegans* (Fonte et al. 2002). The HSP‐16.1, HSP‐16.2, and HSP‐16.48 are orthologs of human αB‐crystallin, and their upregulation was observed in AD brains, as we observed in our study (Alexander et al. 2014). Furthermore, researchers observed that increasing HSP‐16.2 levels in transgenic *C. elegans* reduced fibril formation. However, growing HSP‐16.2 levels could not reduce overall Aβ accumulation (Fonte et al. 2008). We hypothesize that the increase in chaperone expression occurred in response to the expression and accumulation of Aβ peptides that became misfolded and aggregated residues.

In MF, the term relating to ligase activity was also enriched (Figure 4C). The UBC‐13 protein was upregulated (*p* < 0.05) (Figure 5I) in the *dvIs2* (CL2006), being the human ortholog of ubiquitin‐conjugating enzyme E2 N (UBE2N). UBE2N is active in ubiquitination processes, in addition to defining where and how a protein target is modified by ubiquitination (Stewart et al. 2016). Ubiquitin is a protein that targets and directs other proteins to the proteasome system, which is critical for neuronal proteostasis (Chocron et al. 2022). Around 80 to 90% of protein degradation occurs through the ubiquitination pathways, an essential system for the degradation of misfolded proteins (Lin et al. 2024). Individuals with AD and the AD model in *C. elegans* present upregulation of UBE2N (Zhang, Jia, et al. 2024). In addition to increased UBE2N levels in AD individuals, mouse and monkey models showed increased aggregation and formation of Aβ dimers due to elevated UBE2N, contributing to cognitive declines (Zhang, Jia, et al. 2024). In general, increased levels of UBE2N prevent the clearance of Aβ oligomers (Zhang, Jia, et al. 2024). We believe this is further evidence that the AD model in *C. elegans* has a strong correlation with findings in humans, reinforcing its validity in AD studies.

Another term enriched by KEGG, pyruvate metabolism, showed changes in the *dvIs2* (CL2006) (Figure 4D). The malate dehydrogenase 2 (MDH‐2) protein, a human ortholog of MDH1‐2 isoforms, was downregulated in the *C. elegans* AD model (*p* < 0.05) (Figure 5J). MDH catalyzes the conversion of malate to oxaloacetate using the essential NAD+/NADH in the citric acid cycle (Peterson et al. 2024). AD individuals also showed downregulation of the MDH protein in a transcriptomic study (Jia et al. 2023). Furthermore, these findings are consistent with the analysis of human datasets used in our research, which also observed the downregulation of MDH isoforms. MDH deficiency in AD can cause neuronal development dysfunctions and correlates with individuals' cognitive decline (Broeks et al. 2019; Jia et al. 2023).

Enolase 1 (ENOL‐1) is upregulated (*p* < 0.05) (Figure 5K) in *dvIs2* (CL2006) and was enriched in KEGG (Figure 4D). Enolase acts in carbon metabolism, specifically in glucose metabolism, converting 2‐phosphoglycerate to phosphoenolpyruvate, and has other biological functions (Butterfield and Lange 2009). Studies have identified high levels of enolase in individuals with early‐onset and non‐early‐onset AD human brains, attributing its increase to processes of excitotoxicity, hypoxia, and/or oxidative stress (Liu, Li, et al. 2024). The *dvIs2* (CL2006) presents a high level of superoxide mediated by the aggregation and toxicity of the Aβ peptide (Diomede et al. 2013). The upregulation of ENOL‐1, which has been proven to be related to oxidative stress, might be involved in the mechanisms of ROS increases in this strain (Castegna et al. 2002; Sultana and Butterfield 2024; Tang et al. 2022).

The glutamate‐oxaloacetate transaminase (GOT) protein was also differentially expressed in the *dvIs2* (CL2006). In *C. elegans* , the GOT‐2.2 protein (*p* < 0.05) was downregulated (Figure 5L). GOT‐2.2 is the human ortholog of GOT‐2, an essential enzyme in carbon metabolism for energy metabolism and cellular respiration, as it transfers the amino group from glutamate to form aspartate (Rumping et al. 2020). Glutamate excitotoxicity is one of the pathogeneses of AD, and it has been observed that GOT can reduce Aβ levels in other animal models for AD (Li et al. 2023). Furthermore, GOT increased the number of hippocampal neurons and synapses (Li et al. 2023). Therefore, we believe that the *dvIs2* (CL2006) can be applied to better unravel the relationships between GOT content and increased Aβ levels in the AD model in *C. elegans* . Furthermore, we hypothesize that GOT‐2.2 downregulation may contribute to the aggregation phenotypes observed in the model.

**FIGURE 6:**
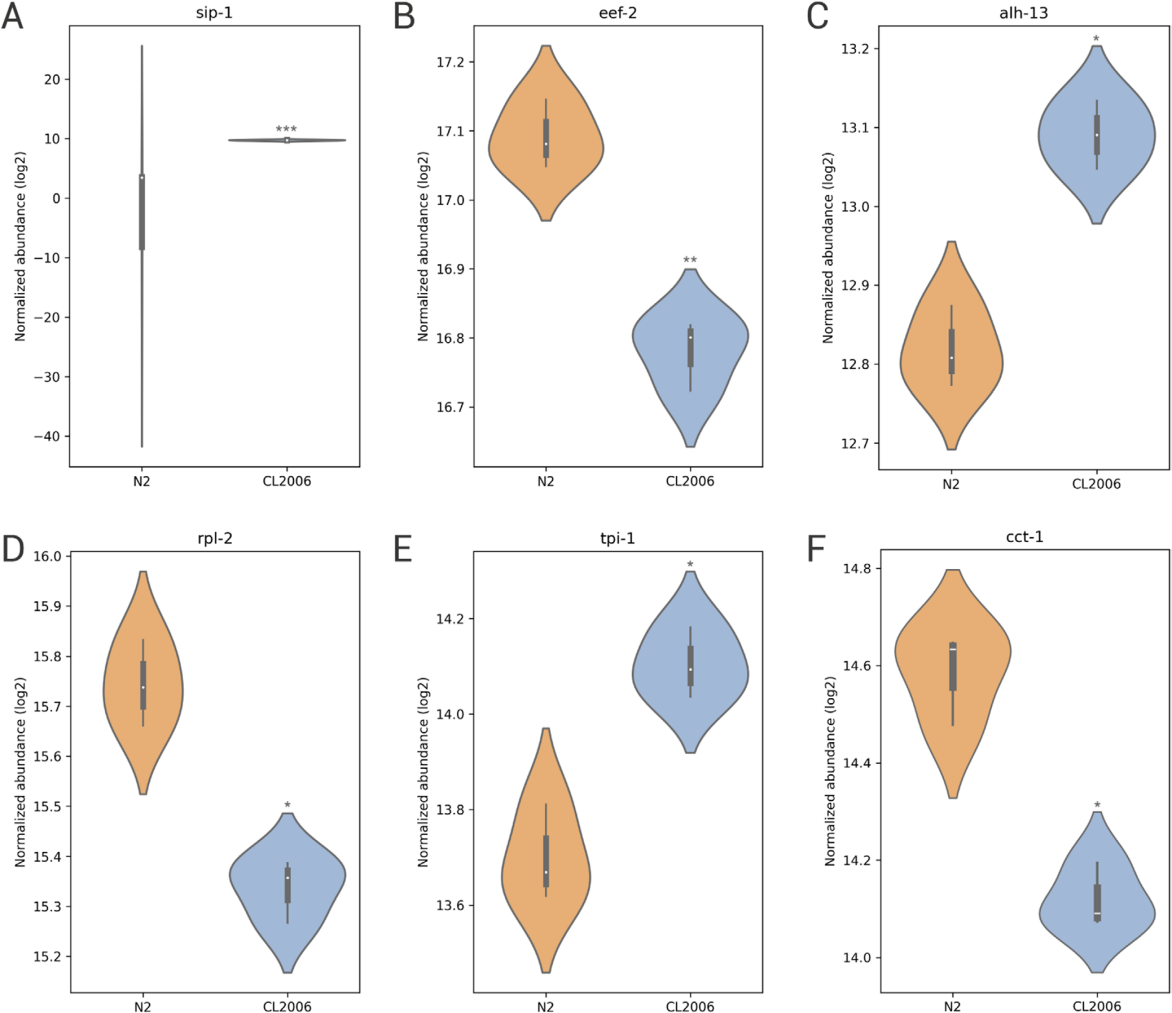
Violin plot visualization of protein expression in N2 wild‐type and *dvls2* (CL2006) by normalized abundance. (A) Stress‐induced protein 1 (SIP‐1). (B) Eukaryotic elongation factor 2 (EEF‐2). (C) Alhambra (ALH‐13). (D) Ribosomal protein L 2 (RPL‐2). (E) Triosephosphate isomerase 1 (TPI‐1). (F) Chaperonin containing TCP‐1 (CCT‐1). Data are represented as distribution plots, with median and quartiles indicated. Statistical significance was determined by a t‐test followed by the Benjamini‐Hochberg procedure (false discovery rate). *p < 0.05, **p < 0.01, ***p < 0.001.

In Reactome, we identified pathways related to tyrosine kinase receptor signaling (Figure 4E). The protein stress‐induced protein 1 (SIP‐1), a human ortholog of αB‐crystallin, was upregulated (*p* < 0.001) in the *C. elegans* AD model (Figure 6A). As in our study, a mouse model for AD also observed increased levels of the αB‐crystallin ortholog in a proteomic study (Do Carmo et al. 2018). αB‐crystallin is a small heat shock protein whose expression is increased through oxidative stress generated by misfolded proteins. Furthermore, αB‐crystallin prevents the aggregation of misfolded proteins such as Aβ (Arrigo et al. 2007).

Previous studies have reported that αB‐crystallin inhibits the formation of Aβ fibrils and reduces their toxicity (Dehle et al. 2010). In the OXYS rat model for AD, researchers observed that levels of αB‐crystallin in the prefrontal cortex were not altered; however, the content of phosphorylated αB‐crystallin was increased, consistent with aging and increases in Aβ deposits (Muraleva et al. 2019). In *C. elegans* , SIP‐1 is essential for thermotolerance and stress tolerance, whose deletion decreases the survival rate (Fleckenstein et al. 2015). Furthermore, SIP‐1 fulfills general proteome protection functions and other chaperones (Fleckenstein et al. 2015). We hypothesize that with the increase in proteotoxic stress and the generation of misfolded proteins, there is a need for the action of SIP‐1, in addition to other chaperones mentioned above.

Some molecular changes can be mistakenly considered as being triggered by physiological aging. In our study, we used *C. elegans* at the young L4 larval stage (~44 h), which does not yet exhibit the hallmarks of aging (Son et al. 2019). It is supported by our control group, which did not show the same alterations as the *dvls2* (CL2006) strain. Therefore, we consider that the omic modifications at the protein level observed in our study are properly modulated by the proteotoxicity generated by the accumulation of Aβ in the animals.

Proteins are essential macromolecules for the performance of biological processes. Therefore, they rarely perform tasks in isolation but in a coordinated way when interacting with other proteins (Jha et al. 2022). We infer protein functions and their inter‐protein communications by analyzing the PPI network of AD‐related orthologous genes in *C. elegans* . Therefore, PPI networks were constructed for human DEGs and others to *C. elegans* DEPs according to the FC cutoff and evaluated for their importance in PPI communication. In PPI, nodes represent proteins, and edges represent non‐directed interactions.

A PPI network was constructed using 397 upregulated and 767 downregulated human DEGs associated with AD. The analysis revealed that the human PPI network had 1041 nodes and 5769 edges. Topological properties define the structural properties of complex networks (Cannistraci et al. 2013). The “*hubs*” of the network are defined as the nodes with the highest degree in the system, tending to play an essential role in the PPI network. The top 10 most important *hub* genes (*TP53*, *EFRG*, *HIF1A*, *EEF2*, *MRPL13*, *MRPS7*, *NDUFAB1*, *RPLP0*, *KRAS*, and *BRCA1*) of the PPI network were identified. The analysis of the topological properties of the PPI network also resulted in 10 main centrality measures (*C*_*C*_ and *C*_*B*_) (Table 2).

**TABLE 2:** Top 10 genes with the highest degree (hub genes), *C*_*B*_, and *C*_*C*_ in the AD PPI network of humans.

S. no.	Name	Degree	Name	*C*_*B*_	Name	*C*_*C*_
1	*TP53*	161	*TP53*	174760.1	*TP53*	531.3
2	*EGFR*	98	*EGFR*	83870.5	*EGFR*	482.8
3	*HIF1A*	82	*KRAS*	45121.3	*HIF1A*	460.8
4	***eEF2***	77	*PRKACB*	43161.5	***eEF2***	460.0
5	*MRPL13*	74	*HIF1A*	39367.5	*KRAS*	458.5
6	*MRPS7*	74	***eEF2***	36650.3	*RPLP0*	447.1
7	*NDUFAB1*	70	*BRCA1*	28521.0	*BRCA1*	446.7
8	*RPLP0*	68	*PPARG*	24711.6	*PRKACB*	437.5
9	*KRAS*	64	*NDUFAB1*	22845.6	*MRPL13*	435.5
10	*BRCA1*	62	*COPS5*	22640.3	*PSMC3*	435.5

*Note:* The genes shown in bold were detected in all network topology analyses.

**FIGURE 7:**
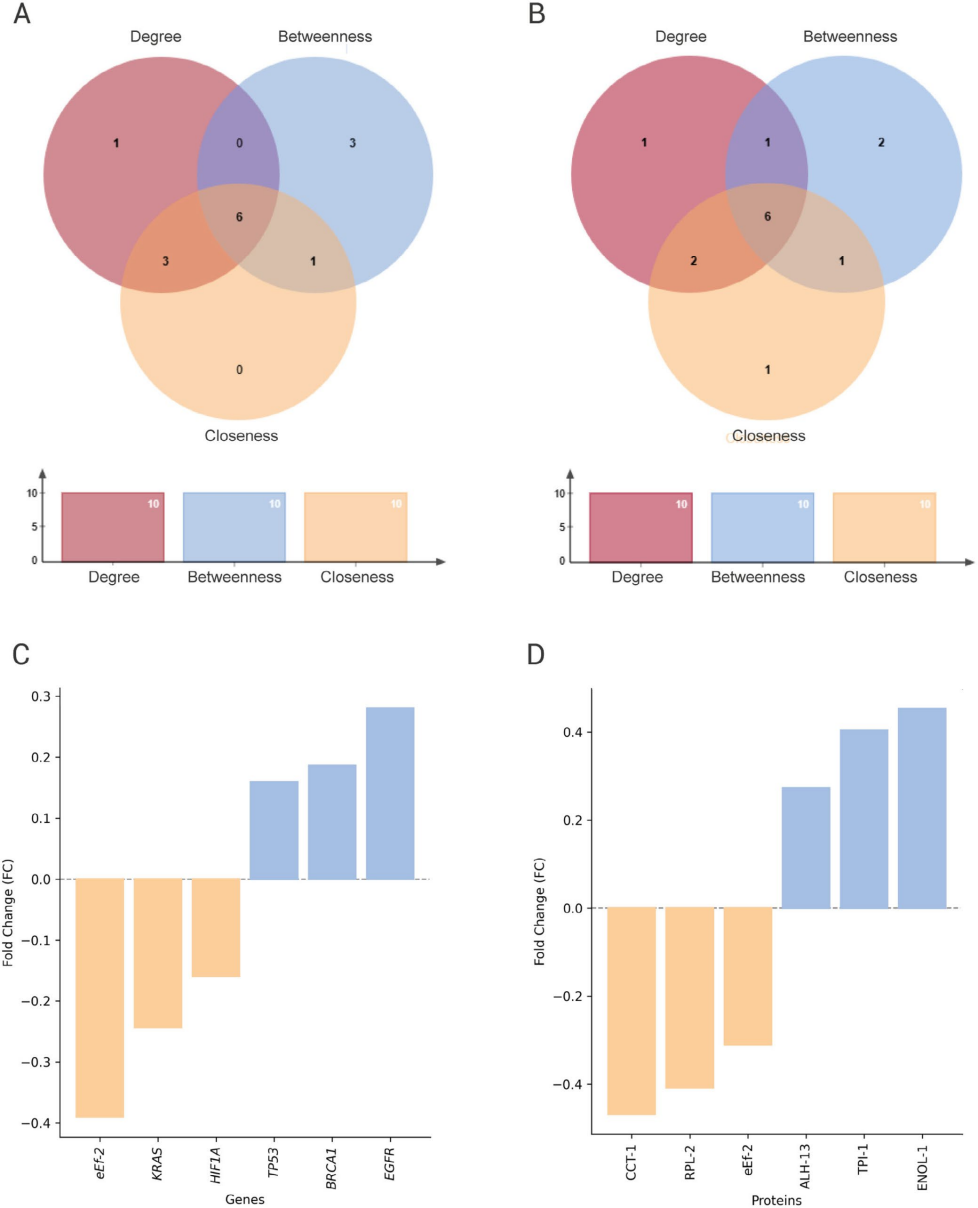
Comparison of differential expression between humans and *C. elegans* . Venn diagram of DEGs in humans (A) and DEPs in *C. elegans* (B). Column graphs (C) and (D) show the direction of regulation (up‐ or downregulated) for the six human DEGs and the corresponding *C. elegans* DEPs, respectively.

To determine the key regulatory human DEGs of the PPI network, the top 10 genes of the three topological characteristics (degree, *C*_*C*_, and *C*_*B*_) were interspersed using the Venn diagram (Figure 7A). Thus, we identified six key regulators of human AD that were common for the three attributes, being the following genes: *TP53*, *EGFR*, *HIF1A*, *EEF2*, *KRAS*, and *BRCA1*, indicating that these genes can regulate and play key roles in AD. *EEF2*, *HIF1A*, and *KRAS* were downregulated, while the other genes were upregulated in the human meta‐analysis dataset (Figure 7C).

**FIGURE 8:**
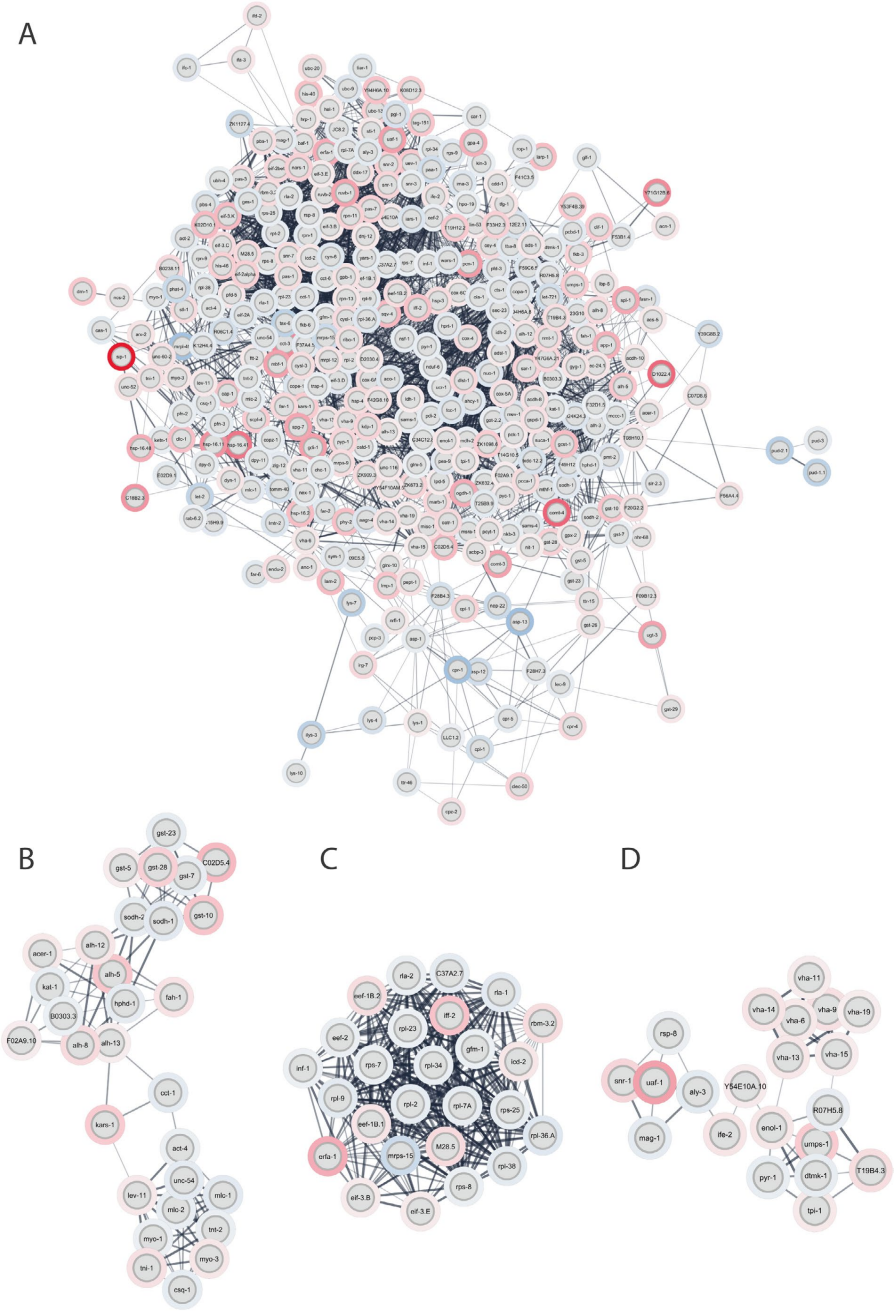
PPI interaction networks from the *dvls2* (CL2006) *C. elegans* AD model. (A) Overall PPI network containing all differentially expressed proteins (DEPs) from the *dvls2* (CL2006) model. (B–D) Subnetworks (modules 3, 1, and 5, respectively) were identified using the MCODE algorithm.

Another PPI network was constructed with the DEPs identified in the proteomics of the *C. elegans dvls2* (CL2006) using the same scheme for human DEGs. The PPI network result showed 356 nodes and 3003 edges (Figure 8A). Therefore, after the analysis, we identified the 10 main “*hub*” proteins according to degree (EEF‐2, ALH‐13, ENOL‐1, RPL‐2, TPI‐1, CTS‐1, RPL‐9, RPL‐23, CCT‐1, and RPS‐8) on the PPI network (Table 3). Topological property analyses were performed similarly, resulting in the top 10 centrality measures (*C*_*C*_ and *C*_*B*_) (Table 3). The essential key regulators of a network are proteins that play central roles in regulating and controlling a biological network (Riaz and Guerinot 2021). Then, the top 10 proteins from three topological features (degree, *C*_*C*_, and *C*_*B*_) were compared using a Venn diagram (Figure 7B). Therefore, six proteins, eEF‐2, ALH‐13, ENOL‐1, RPL‐2, TPI‐1, and CCT‐1, were familiar with the three attributes (Figure 7B).

**TABLE 3:** Top 10 proteins with the highest degree (hub proteins), *C*_*B*_, and *C*_*C*_ in the *dvls2* (CL2006) AD model PPI network of *C. elegans* .

S. no.	Name	Degree	Name	*C*_*B*_	Name	*C*_*C*_
1	**eEF‐2**	78	ENOL‐1	9525.2	**eEF‐2**	199.9
2	ALH‐13	72	ALH‐13	8499.1	ENOL‐1	198.6
3	ENOL‐1	71	**eEF‐2**	6975.5	ALH‐13	197.9
4	RPL‐2	71	T08H10.1	5435.7	RPL‐2	194.2
5	TPI‐1	61	RPL‐2	5068.7	TPI‐1	191.1
6	RPL‐23	60	VHA‐6	5020.9	CCT‐1	188.6
7	RPL‐9	59	PYP‐1	4660.3	RPL‐23	185.9
8	CTS‐1	57	CCT‐1	4375.1	RPL‐9	185.4
9	CCT‐1	56	TPI‐1	4259.0	PYP‐1	185.0
10	RPS‐8	53	HPHD‐1	3932.8	CTS‐1	184.6

*Note:* The genes shown in bold were detected in all network topology analyses.

When a comparison between the regulated nodes of humans and *C. elegans* was made, we found that eEF2, an essential key regulator in the human DEGs network, serves as a key regulator in the orthologous *C. elegans* DEPs network. Furthermore, other genes such as ENOL‐1, although not critical key regulators in human PPI, have been identified in *C. elegans* and have orthologs in humans. All these results indicate that the identified orthologous genes can play a crucial role in elucidating the mechanisms of AD, serving to improve the *dvIs2* (CL2006).

Elongation factor 2 (eEF‐2) is a protein that catalyzes GTP‐dependent ribosomal translocation during translation elongation, where *de novo* protein synthesis is partially mediated by eEF‐2 (Kaul et al. 2011; Gosrani et al. 2020). When eEF‐2 is phosphorylated by the kinase eEF2K, it inhibits its activity and suppresses protein synthesis (Proud 2015). In AD, the molecular mechanisms that regulate cognitive deficits associated with aging are still unclear, with memory and synaptic plasticity dependent on *de novo* protein synthesis (Gosrani et al. 2020). Some researchers observed that the eEF2 protein was downregulated in AD individuals in early stages I–II of the disease (Hernández‐Ortega et al. 2016). Therefore, our study also detected downregulation of eEF‐2 (*p* < 0.01) in the AD model in *C. elegans* (Figure 6B). Furthermore, the eEF‐2 of *C. elegans* has high identity with the human one, as shown by the alignment of the amino acid sequence of the two organisms with 78.74% identity (https://blast.ncbi.nlm.nih.gov/Blast.cgi?CMD=Get&RID=0N4MT66M016).

eEF‐2 is essential and highly conserved between humans and *C. elegans* (Sonenberg and Hinnebusch 2009). From an evolutionary perspective, the structural and functional conservation of eEF‐2 ensures efficient and accurate translation for cellular survival and proper functioning of organisms (Jia et al. 2024). Also, in the evolutionary context, the high conservation of eEF‐2 among eukaryotes exemplifies how proteins essential for central cellular processes are maintained. Furthermore, this evolutionary conservation between humans and *C. elegans* demonstrates the importance of translation as a tightly controlled vital process that sustains cellular function.

In another mouse model for AD, a reduction in protein synthesis in the prefrontal cortex was observed, correlating with decreased levels of eEF2 (Garcia‐Esparcia et al. 2017). The downregulation of eEF‐2 observed in our study may reflect dysfunctions in the *de novo* production of proteins associated with AD. Furthermore, as the hyperphosphorylation of eEF2 present in individuals with AD causes the suppression of protein synthesis (Chi et al. 2025), we propose that it is possible to draw a parallel between the effects of eEF2 downregulation in our model and those found in individuals with eEF2 at normal levels.

An analysis of modules in PPI was performed to help us understand the dynamics of *C. elegans* PPI networks. The modules represent groups of proteins that tend to interact with each other more frequently (Guimarães et al. 2018). Investigating the dynamics of modules can provide valuable insights into adaptability in various circumstances. Therefore, the initial native network was divided into subnets or modules using the MCODE plugin in Cytoscape 3.10.1. The parameters used were the default, that is, “Degree cutoff = 2,” “node score cutoff = 0.2,” “k‐score = 2,” and “max. depth = 100”. MCODE identified 13 modules, where only seven were considered significant with MCODE Score ≥ 5 and generated separate modules from the native network. Interestingly, key regulators were identified within some of the analyzed modules. The modules with key regulators present were module 1 (score: 22.8) with 26 nodes, 286 edges, and the presence of key regulators eEF‐2 and RPL‐2 (Figure 8B); module 3 (score: 8) with 30 nodes and 117 edges with CCT‐1 and ALH‐13 (Figure 8C); and module 5 (score: 5.4) with 21 nodes and 54 edges with ENOL‐1 and TPI‐1 (Figure 8D).

Identifying modules is important as it helps identify functionally cohesive groups within the PPI network. These modules correspond to functional units, such as protein complexes or signaling pathways. The presence of key regulators in some modules reinforces their functional relevance, as these proteins may act as central nodes in the adaptation and stability of the network.

CCT‐1 protein was downregulated (*p* < 0.05) (Figure 6F), while ALH‐13 was upregulated (*p* < 0.05) (Figure 6C). Previously, we identified in other studies the increase of superoxide levels in *dvls2* (CL2006) (Diomede et al. 2013) in response to Aβ accumulation. ALH‐13, a probable delta‐1‐pyrroline‐5‐carboxylate synthase, is responsible for the biosynthesis of proline from glutamate, acting in the cellular redox balance (Yen and Curran 2021). We propose that the increase in ALH‐13 may be related to adaptive responses to redox stress triggered by Aβ accumulation. Other researchers have observed that proline can increase the thermotolerance of *C. elegans* by stabilizing proteins and membranes (Edwards et al. 2015).

The decrease in levels of eEF‐2 and RPL‐2 (*p* < 0.05) (Figure 6D) may reflect the activation of the integrated stress response (ISR), whose conserved mechanism is activated in times of proteotoxic and/or oxidative stress, repressing global protein translation in order to reduce the accumulation of misfolded proteins (Pakos‐Zebrucka et al. 2016). In *C. elegans* , the ISR involves the phosphorylation of eIF2α, thereby reducing the activity of the ribosomal machinery and elongation (Taniuchi et al. 2016). Therefore, one of our hypotheses is that one of the possible mechanisms downregulating these proteins may be linked to adaptive adjustments in translation, prioritizing the promotion of defense proteins such as chaperones (Brostrom and Brostrom 1997; Alagar Boopathy et al. 2022). Mouse models of AD have also shown increased ISR, in addition to postmortem brain tissue (Lockshin and Calakos 2024).

The accumulation of Aβ is capable of triggering mitochondrial dysfunctions, which may help explain the upregulation of the proteins ENOL‐1 (*p* < 0.05) and TPI‐1 (*p* < 0.05) (Figure 6E) observed in *dvls2* (CL2006). Both proteins act in the regulation of glycolysis, where Enolase 1 (ENOL‐1), for example, converts 2P‐glycerate into phosphoenolpyruvate (Didiasova et al. 2019). Triosephosphate isomerase (TPI‐1), in turn, converts dihydroxyacetone phosphate into glyceraldehyde‐3‐phosphate (Myers and Palladino 2023). Oxidative phosphorylation processes may be key regulators of the increase in these protein levels in response to mitochondrial damage (Zong et al. 2024). Therefore, we hypothesize that Aβ‐induced toxicity is capable of increasing glycolytic flux in order to maintain ATP production due to mitochondrial dysfunctions.

Future perspectives for this model include combining *dvls2* (CL2006) with fluorescent reporters to observe mitochondrial function or protein aggregation for high‐resolution and real‐time monitoring of pathological processes. These modifications would enable a closer examination of the neurodegenerative aspects of AD and facilitate a deeper investigation. These improvements would enhance the model's utility for pharmacological screening and mechanism studies. Although *C. elegans dvls2* (CL2006) is shown to be a great experimental model for AD studies, it has certain limitations where improvements are needed. Transgenic strains have shorter lifespans and reduced viability of progeny compared to wild‐type strains, which may pose challenges in maintaining synchronized populations and performing long‐term assays (Tang et al. 2022). Furthermore, *dvls2* (CL2006) has proven to be a powerful tool for accelerating preclinical screening of drug candidates, and this should be further explored.

## 4 Conclusions and Future Perspectives

Comparative analyses between experimental models for studying human diseases are essential for improving the reliability of comparisons and extrapolations made. Therefore, we submitted the AD model in *C. elegans dvls2* (CL2006) to proteomic studies, drawing comparisons with microarray datasets of human individuals with AD and also comparing the findings with other animal models widely used in scientific research for AD.

Functional enrichment highlighted changes in the status of protein refolding and proteins related to muscle processes in the *dvls2* (CL2006) model. In addition, unfolded protein binding and ligase activity were activities presented by these proteins. The metabolism of pyruvate, glucose, and glutamate was also altered in the AD model in *C. elegans* . Human patients and other murine animal models, for example, also presented the same alterations at the protein level. An important class of proteins that act on misfolded proteins, chaperones, was increased in *dvls2* (CL2006) animals, along with proteins related to ubiquitination processes. Another important finding is related to protein synthesis, since the AD model in *C. elegans* presented downregulation of eEF‐2, a protein that catalyzes GTP‐dependent ribosomal translocation during translation elongation, which is also present at low levels in AD patients. Furthermore, eEF‐2 has been shown to be a key regulator in the human AD interaction network and in *C. elegans* , which may inspire further research to study this relationship.

Therefore, our work was able to illuminate previously obscure aspects of the biology of the AD model in *C. elegans* . Understanding the similarities and differences between the biological reality of models for human diseases is crucial for the reliable conduct of the findings. We believe that our study can contribute to future explanations of the use of the AD model *dvls2* (CL2006) in the prospecting of new molecules with the potential to inhibit Aβ peptide aggregation and help in a deeper understanding of disease development.

## Author Contributions

**Iverson Conrado Bezerra:** conceptualization, investigation, writing – original draft, methodology, validation, visualization, formal analysis, data curation. **Emily Raphaely Souza dos Santos:** investigation, writing – original draft, methodology, validation, visualization, writing – review and editing. **Katarine G. Aurista do Nascimento:** investigation, writing – original draft, methodology, validation, visualization, writing – review and editing. **Artur José da Silva:** investigation, writing – original draft, methodology, validation, visualization, writing – review and editing. **Josivan Barbosa de Farias:** investigation, writing – original draft, methodology, validation, writing – review and editing. **Maria Luiza de Lima Vitorino:** investigation, writing – original draft, methodology, writing – review and editing. **Roberto Afonso da Silva:** investigation, writing – original draft, methodology, validation, writing – review and editing, formal analysis. **José Luiz de Lima Filho:** funding acquisition, investigation, writing – review and editing, project administration. **Priscila Gubert:** investigation, funding acquisition, writing – original draft, methodology, validation, writing – review and editing, formal analysis, project administration, data curation, supervision.

## Conflicts of Interest

The authors declare no conflicts of interest.

## Peer Review

The peer review history for this article is available at https://www.webofscience.com/api/gateway/wos/peer‐review/10.1111/jnc.70152.

## Supporting information


**Table S1.** Quality control results of human datasets of AD individuals.
**Table S2.** Orthologous genes in *C. elegans* of proteins detected in proteomics by comparative analysis. 29 upregulated and 24 downregulated DEPs, according to the FC cutoff. Function information came from the Database Alliance of genome resources and the ortholog OrthList2.
**Figure S1.** Transgenicity verification by RT‐qPCR of *dvls2* (CL2006) strain to Aβ gene. (A) Amplification plot of the Aβ amplicon. (B) Melting curve showing the generation of only one amplicon. (C) ΔCq of the Aβ gene. The result was represented as mean ± SD of triplicate.
**Figure S2.** Dynamic range of *dvls2* ( *C. elegans* ) protein abundance across the dataset. Each point represents a quantified protein. Proteins highlighted in yellow indicate those of particular relevance to this study.
**Figure S3.** Enrichment analysis of *dvls2* (CL2006) *C. elegans* DEPs according to FC. (A) Biological process. (B) Cellular component. (C) Molecular function. (D) KEGG Pathways. (E) Reactome pathways.

**Table S3.** The meta‐analysis of AD human genes.

**Table S4.** Human orthologous genes in *C. elegans* identified in Ortholist 2.

**Table S5.** Statistical analysis details.

## Data Availability

The datasets generated and analyzed during the current study are available from the author upon reasonable request.
