# Supplementary material for: Label‐Free Proteomic Profiling of the dvls2 (CL2006) Caenorhabditis elegans Alzheimer's Disease (AD) Model Reveals Conserved Molecular Signatures Shared With the Human AD Brain
Source: J Neurochem. 2025 Jul 13;169(7):e70152. doi: 10.1111/jnc.70152 (PMC12256974; doi:10.1111/jnc.70152)
Supplement: Supplementary file 1 — Table S1. Quality control results of human datasets of AD individuals. Table S2. Orthologous genes in C. elegans of proteins detected in proteomics by comparative analysis. 29 upregulated and 24 downregulated DEPs, according to the FC cutoff. Function information came from the Database Alliance of genome resources and the ortholog OrthList2. Figure S1. Transgenicity verification by RT‐qPCR of dvls2 (CL2006) strain to Aβ gene. (A) Amplification plot of the Aβ amplicon. (B) Melting curve showing the generation of only one amplicon. (C) ΔCq of the Aβ gene. The result was represented as mean ± SD of triplicate. Figure S2. Dynamic range of dvls2 ( C. elegans ) protein abundance across the dataset. Each point represents a quantified protein. Proteins highlighted in yellow indicate those of particular relevance to this study. Figure S3. Enrichment analysis of dvls2 (CL2006) C. elegans DEPs according to FC. (A) Biological process. (B) Cellular component. (C) Molecular function. (D) KEGG Pathways. (E) Reactome pathways. [file JNC-169-0-s004.pdf]

Supplementary materials

# Label-Free Proteomic profiling of the *dvls2* (CL2006) *Caenorhabditis elegans* Alzheimer's Disease (AD) model reveals conserved molecular signatures shared with the human AD brain

Iverson Conrado Bezerra[1,2], Emily Raphaely Souza dos Santos[1], Katarine G. Aurista do Nascimento[1,2], Artur José da Silva[1,2], Josivan Barbosa de Farias[1,2], Maria Luiza de Lima Vitorino[1,2], Roberto Afonso da Silva[1], José Luiz de Lima Filho[1], Priscila Gubert[1,2*]

**Affiliations:**

[1] Keizo Asami Institute, iLIKA, Federal University of Pernambuco, Recife, Brazil.

[2] Graduate Program in Biology Applied to Health, PPGBAS, Federal University of Pernambuco, Recife, Brazil.

* Corresponding author

E-mail address: priscila.gubert@ufpe.br (Priscila Gubert).
**ORCID:** https://orcid.org/0000-0002-3598-8050
+ 55 (81) 2126-8484

**Table S1.** Quality control results of human datasets of AD individuals.

| Datasets ID | Sample_preQC | Samples_postQC | Imputed_Genes | PASS_QC |
|---|---|---|---|---|
| GSE48350 | 253 | 253 | 0 | Yes |
| GSE5281 | 161 | 0 | Yes | Yes |
| GSE36980 | 80 | 80 | 0 | Yes |

**Table S2.** Orthologous genes in *C. elegans* of proteins detected in proteomics by comparative analysis. 29 up-regulated and 24 down-regulated DEPs, according to the FC cutoff. Function information came from the Database Alliance of genome resources and the ortholog OrthList2.

| *C. elegans* gene | Accession | Log2(FC) | Function | Human orthologous | Function |
|---|---|---|---|---|---|
| sip-1 | Q20363 | 6.69 | Enables several functions, including amyloid-beta binding activity; protein homodimerization activity; and unfolded protein binding activity. | CRYAB | Amyloid-beta binding activity; protein homodimerization activity; and unfolded protein binding activity |
| hsp-16.41 | P06581 | 3.44 | Predicted to enable unfolded protein binding activity. | CRYAA | Predicted to enable identical protein binding activity; structural molecule activity; and unfolded protein binding activity. Regulation of macromolecule metabolic process; and tubulin complex assembly |
| hsp-16.2 | P06582 | 2.69 | HSP-16.2 has been shown to interact with intracellular human beta amyloid peptide, a primary component of the extracellular plaques found in Alzheimer's disease; HSP-16.2 is likely to function as a passive ligand temporarily preventing unfolded proteins from aggregating. | CRYAB | Amyloid-beta binding activity; protein homodimerization activity; and unfolded protein binding activity. |

| | | | | | |
|---|---|---|---|---|---|
| hsp-16.1 | P34696 | 2.65 | Used to study neurodegenerative disease. HSP-16.1 is likely to function as a passive ligand temporarily preventing unfolded proteins from aggregating. | CRYAB | Amyloid-beta binding activity; protein homodimerization activity; and unfolded protein binding activity. |
| hsp-16.48 | P02513 | 1.96 | Predicted to enable unfolded protein binding activity. Involved in determination of adult lifespan and response to heat. Predicted to be active in cytoplasm and nucleus | CRYAB | Amyloid-beta binding activity; protein homodimerization activity; and unfolded protein binding activity. |
| lam-2 | Q18823 | 1.58 | Involved in positive regulation of locomotion. Predicted to be active in the basement membrane. | LAMC1 | An extracellular matrix structural constituent. Involved in several processes, including extracellular matrix disassembly; hemidesmosome assembly; and substrate adhesion-dependent cell spreading. |

| | | | | | |
|---|---|---|---|---|---|
| kars-1 | Q95ZQ3 | 1.17 | Predicted to enable lysine-tRNA ligase activity and tRNA binding activity. Involved in several processes, including determination of adult lifespan; positive regulation of nematode larval development; and regulation of macromolecule metabolic process. Predicted to be part of aminoacyl-tRNA synthetase multienzyme complex. | KARS1 | Enables ATP:ADP adenylyltransferase activity; lysine-tRNA ligase activity; and protein homodimerization activity. Involved in several processes, including ERK1 and ERK2 cascade; diadenosine tetraphosphate biosynthetic process; and lysyl-tRNA aminoacylation. |
| snr-2 | P91918 | 1.10 | Predicted to enable snRNP binding activity. Predicted to be involved in mRNA splicing, via spliceosome. Predicted to be located in cytosol and nucleus. Predicted to be part of spliceosomal complex and spliceosomal snRNP complex. Predicted to be active in cytoplasm. Is expressed in gonad. | SNURF,SNRPN | Predicted to enable snRNP binding activity. Predicted to be involved in mRNA splicing, via spliceosome. |
| ubc-13 | Q95XX0 | 1.07 | Contributes to ubiquitin-protein transferase activity. Involved in protein ubiquitination and regulation of protein localization to cell surface. | UBE2N,UBE2NL | Predicted to enable ubiquitin conjugating enzyme activity. |

| | | | | | |
|---|---|---|---|---|---|
| nars-1 | Q19722 | 1.01 | Predicted to enable asparagine-tRNA ligase activity. Involved in translation. | NARS1 | Enables CCR3 chemokine receptor binding activity; asparagine-tRNA ligase activity; and protein dimerization activity. Involved in asparaginyl-tRNA aminoacylation; cell migration; and cerebral cortex development. |
| pas-7 | Q09583 | 1.00 | Enables identical protein binding activity. Predicted to be involved in ubiquitin-dependent protein catabolic process. | PSMA3 | Enables ubiquitin protein ligase binding activity. Involved in regulation of endopeptidase activity. |
| stl-1 | H2FLJ1 | 0.74 | Predicted to be involved in mitochondrion organization. | STOML2 | Enables GTPase binding activity; T cell receptor binding activity; and cardiolipin binding activity. Involved in intracellular calcium ion homeostasis; mitochondrion organization; and protein complex oligomerization. |
| mrps-9 | P34388 | 0.70 | Predicted to enable RNA binding activity. Predicted to be a structural constituent of ribosome. | MRPS9 | Enables RNA binding activity. Predicted to be involved in translation. |

| | | | | | |
|---|---|---|---|---|---|
| unc-70 | E0AHA7;G5EG85;Q9U9J8;S6FN00 | 0.67 | Predicted to enable actin filament binding activity. Involved in several processes, including dendrite development | SPTB | Enables actin filament binding activity and ankyrin binding activity. Involved in modification of postsynaptic actin cytoskeleton. Part of the protein-containing complex. Is active in glutamatergic synapse. |
| fars-1 | Q86B36;Q9GYS8 | 0.55 | Predicted to enable phenylalanine-tRNA ligase activity. Involved in translation. Predicted to be part of phenylalanine-tRNA ligase complex | FARSA | Enables phenylalanine-tRNA ligase activity. Involved in phenylalanyl-tRNA aminoacylation and protein heterotetramerization. Located in the membrane. Part of phenylalanine-tRNA ligase complex. |
| hsp-25 | Q17849;Q86GU1;Q5H9M9 | 0.53 | Predicted to enable unfolded protein binding activity. Predicted to be involved in protein refolding and response to heat. Located in M band and striated muscle dense body. Is expressed in body wall musculature; gonad; and pharynx | HSPB1 | Enables several functions, including protein folding chaperone; protein homodimerization activity; and protein kinase binding activity. Involved in several processes, including anterograde axonal protein transport; chaperone-mediated protein folding; and positive regulation of endothelial cell migration. |

| | | | | | |
|---|---|---|---|---|---|
| ant-1.4 | G5EFU2;G5EFW8 | 0.50 | Predicted to enable ATP:ADP antiporter activity. Predicted to be involved in negative regulation of mitochondrial outer membrane permeabilization involved in apoptotic signaling pathway. | SLC25A6 | Predicted to enable ATP:ADP antiporter activity. Predicted to be involved in negative regulation of mitochondrial outer membrane permeabilization involved in apoptotic signaling pathway. |
| alh-12 | Q7Z1Q2 | 0.47 | Predicted to enable 4-trimethylammoniobutyraldehyde dehydrogenase activity and aminobutyraldehyde dehydrogenase (NAD+) activity | ALDH9A1 | Enables oxidoreductase activity, acting on the aldehyde or oxo group of donors, NAD or NADP as acceptor and small molecule binding activity. Involved in aldehyde metabolic process and protein homotetramerization. |
| acs-19 | Q18496;Q65ZB8 | 0.45 | Involved in positive regulation of proteasomal ubiquitin-dependent protein catabolic process and regulation of response to oxidative stress. | ACSS2 | Enables acetate-CoA ligase activity. Involved in lipid biosynthetic process. Located in cytosol and nucleus. Implicated in orofacial cleft. Biomarker of lymphangioleiomyomatosis and morbid obesity. |

| | | | | | |
|---|---|---|---|---|---|
| enol-1 | Q27527 | 0.45 | Human ortholog(s) of this gene implicated in Alzheimer's disease and prostate cancer. | ENO2 | Predicted to enable enzyme binding activity and phosphopyruvate hydratase activity. Predicted to be involved in glycolytic process. |
| myo-3 | P12844 | 0.45 | Enables cytoskeletal motor activity. Involved in locomotory behavior and positive regulation of ovulation. | MYH | Predicted to enable actin filament binding activity and microfilament motor activity |
| vha-2 | C0HLB3;C0HLB4 | 0.45 | Predicted to enable proton-transporting ATPase activity, rotational mechanism | ATP6V0C | Enables ubiquitin protein ligase binding activity. Involved in positive regulation of Wnt signaling pathway |
| vha-13 | Q9XW92 | 0.41 | Predicted to enable proton-transporting ATPase activity, rotational mechanism. | ATP6V1A | Predicted to enable proton-transporting ATPase activity, rotational mechanism. Involved in cellular response to increased oxygen levels and intracellular iron ion homeostasis |
| lbp-5 | O01814 | 0.40 | Enables fatty acid binding activity. Predicted to be involved in fatty acid transport. | FABP7, PMP2 | Enables cholesterol binding activity and fatty acid binding activity. Predicted to be involved in fatty acid transport. Predicted to act upstream of or within membrane organization. |

| | | | | | |
|---|---|---|---|---|---|
| hrp-1 | Q22037 | 0.40 | This protein is a component of ribonucleosomes. Overexpression gradually increases telomere length, leading to increase lifespan. | HNRNPA1 | Enables identical protein binding activity; nucleic acid binding activity; and protein domain specific binding activity. |
| dnj-12 | O45502 | 0.39 | Enables ATPase activator activity. Predicted to be involved in protein refolding. | DNAJA1, DNAJA4 | Enables protein-folding chaperone binding activity and unfolded protein binding activity. |
| ftt-2 | Q20655 | 0.38 | Enables DNA-binding transcription factor binding activity. | YWHAB, YWHAZ, YWHAQ | Enzyme inhibitor activity; histone deacetylase binding activity; and phosphoserine residue binding activity. Involved in cytoplasmic sequestering of protein and negative regulation of G protein-coupled receptor signaling pathway. |
| atn-1 | Q23158;H2L2C9;Q9XVU8;H2L2C8 | 0.34 | Predicted to enable actin filament binding activity. Predicted to be involved in actin cytoskeleton organization and muscle cell development. | ACTN1, ACTN2, ACTN3, ACTN4 | Enables several functions, including cytoskeletal protein binding activity; nuclear receptor coactivator activity; and protein homodimerization activity. |

| | | | | | |
|---|---|---|---|---|---|
| adsl-1 | Q21774 | 0.34 | Predicted to be involved in 'de novo' AMP biosynthetic process. | ADSL | Enables (S)-2-(5-amino-1-(5-phospho-D-ribosyl)imidazole-4-carboxamido) succinate lyase (fumarate-forming) activity; N6-(1,2-dicarboxyethyl)AMP AMP-lyase (fumarate-forming) activity; and identical protein binding activity. |
| lec-2 | Q20684;G5EGB1 | -0.27 | Predicted to enable carbohydrate binding activity and galactoside binding activity. Located in the membrane raft and plasma membrane. | LGALS8 | Enables carbohydrate binding activity and integrin binding activity. Involved in cellular response to virus; lymphatic endothelial cell migration; and xenophagy. |
| acly-2 | P90731;G5EFT2;Q2Q3B5;Q2Q3B9;Q2Q3C3;Q2Q3D1;Q2Q3D2;Q2Q3B4 | -0.28 | Predicted to enable ATP citrate synthase activity. Predicted to be involved in acetyl-CoA biosynthetic process and fatty acid biosynthetic process. | ACLY | Enables ATP citrate synthase activity. Involved in acetyl-CoA biosynthetic process and carboxylic acid metabolic process. Located in cytosol and nucleoplasm. |
| myo-1 | P02567 | -0.28 | A structural constituent of muscle. Involved in muscle contraction and nematode pharyngeal pumping. | MYH78 | Predicted to enable actin filament binding activity and microfilament motor activity. Predicted to be involved in regulation of CAMKK-AMPK signaling cascade |

| | | | | | |
|---|---|---|---|---|---|
| rsp-8 | Q9XTZ2 | -0.29 | Predicted to enable RNA binding activity. Predicted to be involved in mRNA splicing, via spliceosome and positive regulation of mRNA splicing, via spliceosome. | TRA2A | Enables RNA binding activity. Involved in mRNA splicing, via spliceosome. Located in nucleolus and nucleoplasm. |
| got-2.2 | Q17994 | -0.30 | Predicted to enable L-aspartate:2-oxoglutarate aminotransferase activity. Predicted to be involved in aspartate catabolic process. | GOT2 | Enables L-aspartate:2-oxoglutarate aminotransferase activity. Involved in several processes, including aspartate catabolic process; malate-aspartate shuttle; and response to ethanol. |
| eef-2 | P29691 | -0.31 | Predicted to enable GTPase activity; ribosome binding activity; and translation elongation factor activity. Involved in defense response to Gram-negative bacterium and negative regulation of gene expression. | EFF2 | Enables several functions, including GTPase activity; ribosome binding activity; and translation elongation factor activity. Involved in positive regulation of translation and translational elongation. |
| kat-1 | Q22100 | -0.33 | Predicted to enable acetyl-CoA C-acetyltransferase activity. | ACAT1 | Enables acetyl-CoA C-acetyltransferase activity; cholesterol O-acyltransferase activity; and potassium ion binding activity. Involved in isoleucine catabolic process; ketone body catabolic process; and nucleoside phosphate metabolic process. |

| | | | | | |
|---|---|---|---|---|---|
| mdh-2 | O02640 | -0.38 | Enables L-malate dehydrogenase (NAD+) activity. Involved in malate metabolic process. Located in mitochondrion | MDH2 | Enables L-malate dehydrogenase (NAD+) activity. Involved in malate metabolic process; malate-aspartate shuttle; and tricarboxylic acid cycle. |
| gst-23 | P91505 | -0.39 | Predicted to enable glutathione transferase activity. Predicted to be involved in glutathione metabolic process. Predicted to be active in cytosol. | GSTP1 | Enables several functions, including anion binding activity; dinitrosyl-iron complex binding activity; and glutathione peroxidase activity. |
| dpy-11 | G5EC91 | -0.40 | Enables protein-disulfide reductase activity. Involved in several processes, including cuticle development involved in collagen and cuticulin-based cuticle molting cycle; post-embryonic body morphogenesis; and protein folding. | TMX1 | Enables enzyme inhibitor activity; protein disulfide isomerase activity; and protein-disulfide reductase activity |
| rpl-2 | Q9XVF7 | -0.40 | | | |
| csq-1 | Q20203 | -0.42 | Enables calcium ion binding activity. Involved in calcium ion homeostasis. Located in several cellular components, including apical plasma membrane; nucleus; and sarcolemma. | CASQ1 | Enables calcium ion binding activity and identical protein binding activity. Involved in protein polymerization and regulation of calcium ion transport. |

| | | | | | |
|---|---|---|---|---|---|
| fkb-6 | O45418 | -0.43 | Enables Hsp90 protein binding activity and peptidyl-prolyl cis-trans isomerase activity. | FKBP4 | Enables heat shock protein binding activity and peptidyl-prolyl cis-trans isomerase activity. Involved in chaperone-mediated protein folding. |
| yars-1 | G5ED95 | -0.43 | Predicted to enable ATP binding activity and tyrosine-tRNA ligase activity. Predicted to be involved in tyrosyl-tRNA aminoacylation. | YARS1, YARS2 | Involved in mitochondrial tyrosyl-tRNA aminoacylation. |
| ola-1 | P91917 | -0.44 | Predicted to enable ATP hydrolysis activity. Involved in behavioral response to starvation; thermosensory behavior; and thermotaxis. Located in endoplasmic reticulum | OLA1 | Enables ATP binding activity and ATP hydrolysis activity. Involved in ATP metabolic process. Located in centrosome and cytosol. |
| pcp-3 | O02252 | -0.46 | Predicted to enable dipeptidyl-peptidase activity. Predicted to be involved in proteolysis. Located in membrane raft | PRSS16 | Predicted to enable dipeptidyl-peptidase activity. Predicted to be involved in proteolysis. Located in endosome and lysosome. |

| | | | | | |
|---|---|---|---|---|---|
| unc-54 | P02566 | -0.48 | Enables actin filament binding activity and microfilament motor activity. A structural constituent of muscle. Involved in several processes, including egg-laying behavior; muscle contraction; and skeletal muscle myosin thick filament assembly. | MYH7 | Enables microfilament motor activity. Involved in several processes, including regulation of heart contraction; regulation of skeletal muscle contraction; and striated muscle contraction. |
| cas-1 | Q95YA9 | -0.48 | Predicted to enable actin binding activity and adenylate cyclase binding activity. Predicted to be involved in actin filament organization; cAMP-mediated signaling; and cell morphogenesis. | CAP1 | Predicted to enable actin binding activity and adenylate cyclase binding activity. Predicted to be involved in actin filament organization; cAMP-mediated signaling; and cell morphogenesis. |
| copa-1 | Q9N4H7 | -0.52 | Predicted to enable structural molecule activity. Predicted to be involved in Golgi vesicle transport and intracellular protein transport. Predicted to be located in COPI-coated vesicle membrane and Golgi membrane | COPA | Predicted to enable mRNA binding activity. Acts upstream of or within pancreatic juice secretion. Located in cytoplasm; extracellular space; and growth cone |
| eif-3.D | P30642 | -0.56 | Predicted to enable translation initiation factor activity. Predicted to be involved in translational initiation. Predicted to be located in cytoplasm. | EIF3D | Enables mRNA cap binding activity. Contributes to translation initiation factor activity. |

| | | | | | |
|---|---|---|---|---|---|
| ubc-9 | Q95017 | -0.63 | Enables RNA polymerase II-specific DNA-binding transcription factor binding activity; SAM domain binding activity; and SUMO conjugating enzyme activity. | UBE2I | Enables several functions, including RING-like zinc finger domain binding activity; SUMO conjugating enzyme activity; and small protein activating enzyme binding activity. |
| ima-3 | Q19969 | -0.66 | Enables DNA-binding transcription factor binding activity; nuclear import signal receptor activity; and nuclear localization sequence binding activity. | KPNA4 | Enables nuclear import signal receptor activity and nuclear localization sequence binding activity. Involved in NLS-bearing protein import into nucleus. |
| paa-1 | Q09543 | -1.04 | Enables DEAD/H-box RNA helicase binding activity. Involved in embryo development. Located in P granule; microtubule cytoskeleton; and nuclear envelope. | PPP2R1A, PPP2R1B | Enables protein antigen binding activity and protein heterodimerization activity |
| let-2 | P17140 | -1.37 | An extracellular matrix structural constituent conferring tensile strength. Involved in several processes, including embryo development; gonad morphogenesis; and regulation of distal tip cell migration. Located in basement membrane | COL4A5 | An extracellular matrix structural constituent conferring tensile strength. Predicted to be involved in extracellular matrix organization. |

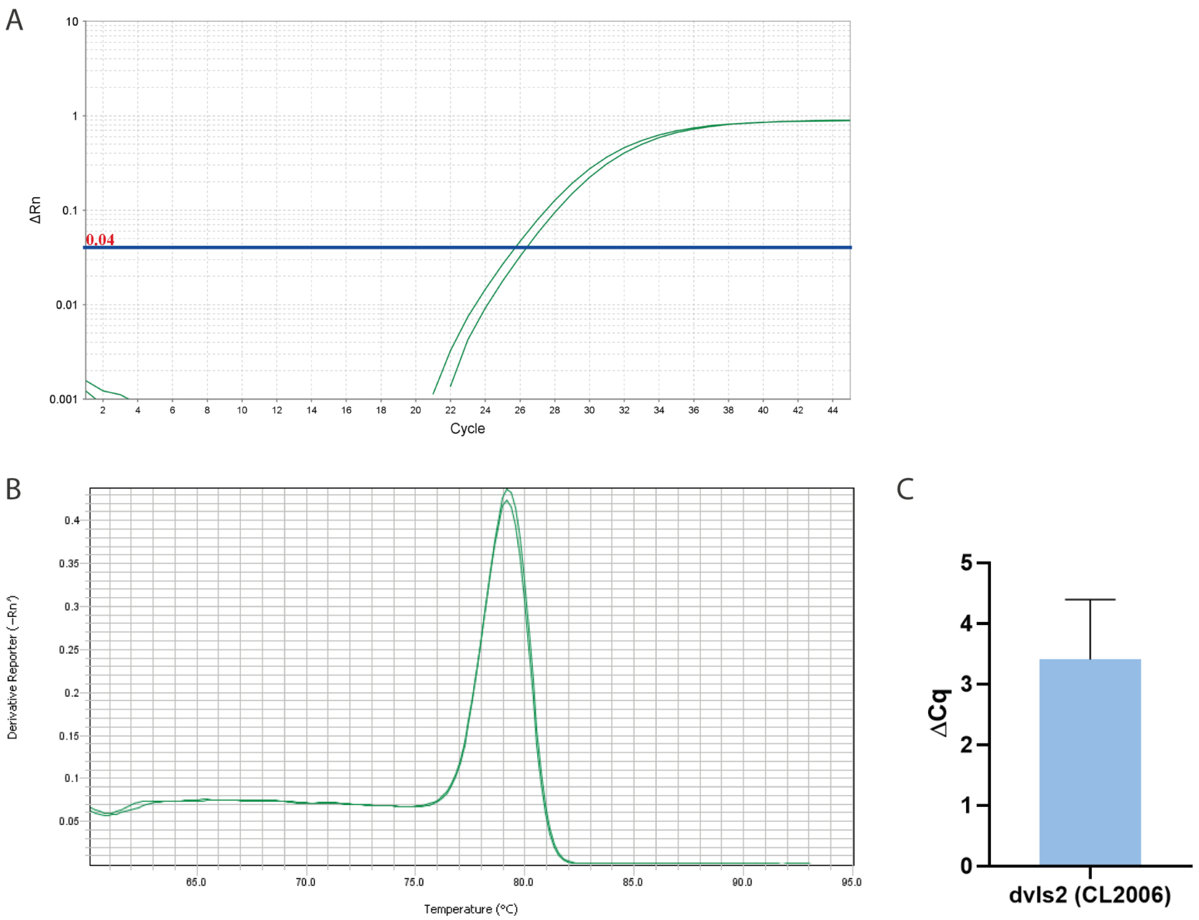


**Figure S1. Transgenicity verification by RT-qPCR of *dvls2* (CL2006) strain to Aβ gene.** (A) Amplification plot of the Aβ amplicon. (B) Melting curve showing the generation of only one amplicon. (C) ΔCq of the Aβ gene. The result was represented as mean ± SD of triplicate.

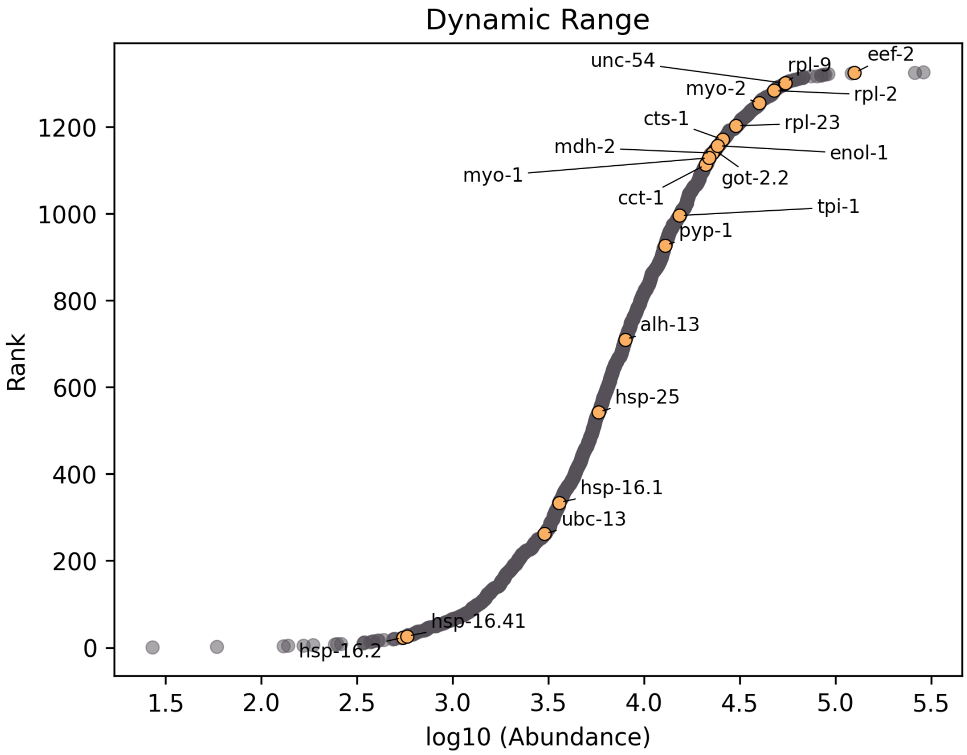


**Figure S2. Dynamic range of *dvls2* (C. elegans) protein abundance across the dataset.** Each point represents a quantified protein. Proteins highlighted in yellow indicate those of particular relevance to this study.

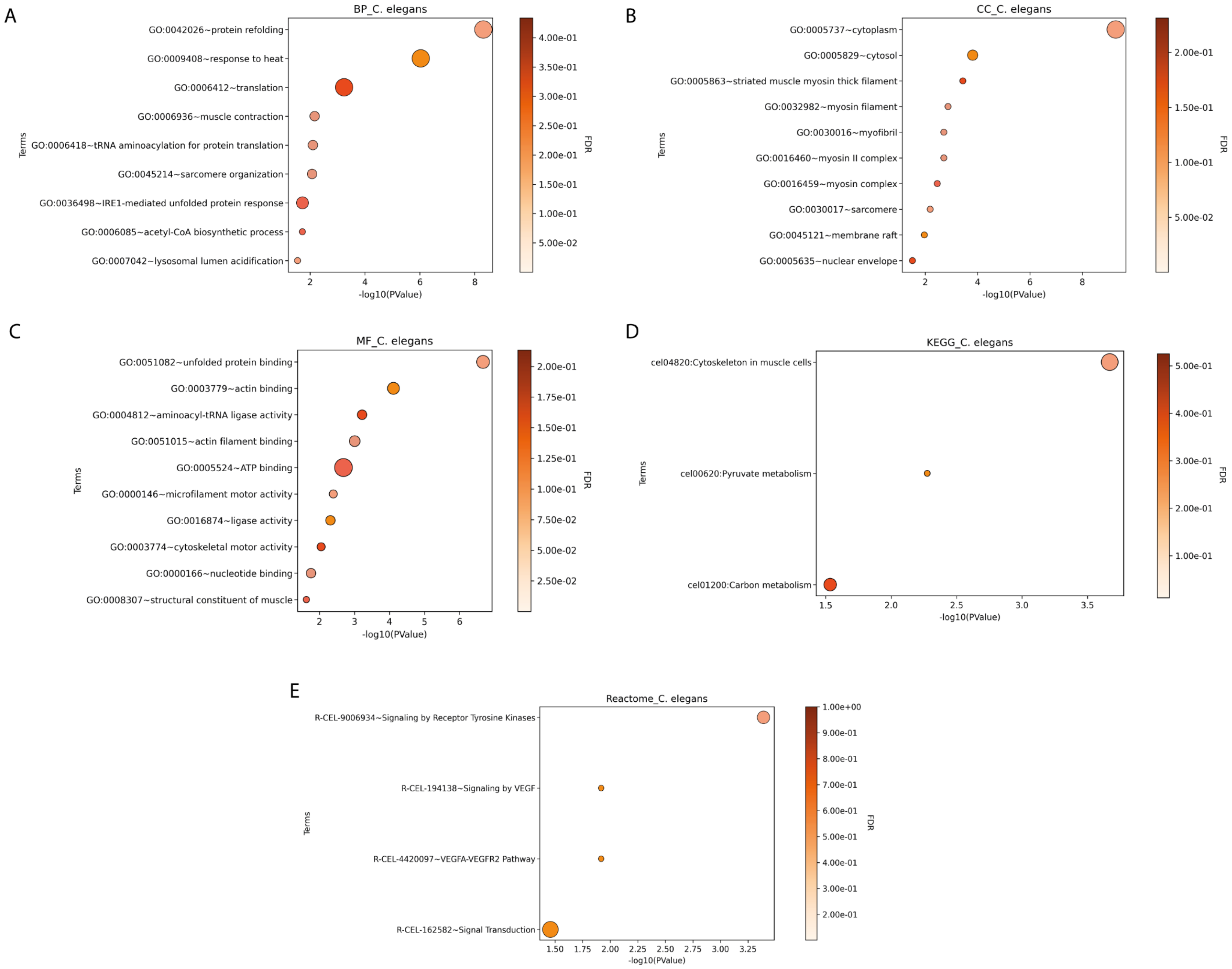

A
BP_C. elegans
GO:0042026~protein refolding
GO:0009408~response to heat
GO:0006412~translation
GO:0006936~muscle contraction
GO:0006418~tRNA aminoacylation for protein translation
GO:0045214~sarcomere organization
GO:0036498~IRE1-mediated unfolded protein response
GO:0006085~acetyl-CoA biosynthetic process
GO:0007042~lysosomal lumen acidification
Terms
-log10(PValue)
2 4 6 8
FDR
4.00e-01 3.50e-01 3.00e-01 2.50e-01 2.00e-01 1.50e-01 1.00e-01 5.00e-02
B
CC_C. elegans
GO:0005737~cytoplasm
GO:0005829~cytosol
GO:0005863~striated muscle myosin thick filament
GO:0032982~myosin filament
GO:0030016~myofibril
GO:0016460~myosin II complex
GO:0016459~myosin complex
GO:0030017~sarcomere
GO:0045121~membrane raft
GO:0005635~nuclear envelope
Terms
-log10(PValue)
2 4 6 8
FDR
2.00e-01 1.50e-01 1.00e-01 5.00e-02
C
MF_C. elegans
GO:0051082~unfolded protein binding
GO:0003779~actin binding
GO:0004812~aminoacyl-tRNA ligase activity
GO:0051015~actin filament binding
GO:0005524~ATP binding
GO:0000146~microfilament motor activity
GO:0016874~ligase activity
GO:0003774~cytoskeletal motor activity
GO:0000166~nucleotide binding
GO:0008307~structural constituent of muscle
Terms
-log10(PValue)
2 3 4 5 6
FDR
2.00e-01 1.75e-01 1.50e-01 1.25e-01 1.00e-01 7.50e-02 5.00e-02 2.50e-02
D
KEGG_C. elegans
cel04820:Cytoskeleton in muscle cells
cel00620:Pyruvate metabolism
cel01200:Carbon metabolism
Terms
-log10(PValue)
1.5 2.0 2.5 3.0 3.5
FDR
5.00e-01 4.00e-01 3.00e-01 2.00e-01 1.00e-01
E
Reactome_C. elegans
R-CEL-9006934~Signaling by Receptor Tyrosine Kinases
R-CEL-194138~Signaling by VEGF
R-CEL-4420097~VEGFA-VEGFR2 Pathway
R-CEL-162582~Signal Transduction
Terms
-log10(PValue)
1.50 1.75 2.00 2.25 2.50 2.75 3.00 3.25
FDR
1.00e+00 9.00e-01 8.00e-01 7.00e-01 6.00e-01 5.00e-01 4.00e-01 3.00e-01 2.00e-01

**Figure S3. Enrichment analysis of dvls2 (CL2006) *C. elegans* DEPs according to FC.** (A) Biological process. (B) Cellular component. (C) Molecular function. (D) KEGG Pathways. (E) Reactome pathways.
